# Pitfalls of machine learning models for protein–protein interaction networks

**DOI:** 10.1093/bioinformatics/btae012

**Published:** 2024-01-10

**Authors:** Loïc Lannelongue, Michael Inouye

**Affiliations:** Cambridge Baker Systems Genomics Initiative, Department of Public Health and Primary Care, University of Cambridge, CB2 0BB Cambridge, United Kingdom; British Heart Foundation Cardiovascular Epidemiology Unit, Department of Public Health and Primary Care, University of Cambridge, CB2 0BB Cambridge, United Kingdom; Victor Phillip Dahdaleh Heart and Lung Research Institute, University of Cambridge, CB2 0BB Cambridge, United Kingdom; Health Data Research UK Cambridge, Wellcome Genome Campus and University of Cambridge, Cambridge, United Kingdom; Cambridge Baker Systems Genomics Initiative, Department of Public Health and Primary Care, University of Cambridge, CB2 0BB Cambridge, United Kingdom; British Heart Foundation Cardiovascular Epidemiology Unit, Department of Public Health and Primary Care, University of Cambridge, CB2 0BB Cambridge, United Kingdom; Victor Phillip Dahdaleh Heart and Lung Research Institute, University of Cambridge, CB2 0BB Cambridge, United Kingdom; Health Data Research UK Cambridge, Wellcome Genome Campus and University of Cambridge, Cambridge, United Kingdom; Cambridge Baker Systems Genomics Initiative, Baker Heart and Diabetes Institute, Melbourne, 3004 Victoria, Australia; British Heart Foundation Centre of Research Excellence, University of Cambridge, CB2 0BB Cambridge, United Kingdom

## Abstract

**Motivation:**

Protein–protein interactions (PPIs) are essential to understanding biological pathways as well as their roles in development and disease. Computational tools, based on classic machine learning, have been successful at predicting PPIs *in silico*, but the lack of consistent and reliable frameworks for this task has led to network models that are difficult to compare and discrepancies between algorithms that remain unexplained.

**Results:**

To better understand the underlying inference mechanisms that underpin these models, we designed an open-source framework for benchmarking that accounts for a range of biological and statistical pitfalls while facilitating reproducibility. We use it to shed light on the impact of network topology and how different algorithms deal with highly connected proteins. By studying functional genomics-based and sequence-based models on human PPIs, we show their complementarity as the former performs best on lone proteins while the latter specializes in interactions involving hubs. We also show that algorithm design has little impact on performance with functional genomic data. We replicate our results between both human and *S. cerevisiae* data and demonstrate that models using functional genomics are better suited to PPI prediction across species. With rapidly increasing amounts of sequence and functional genomics data, our study provides a principled foundation for future construction, comparison, and application of PPI networks.

**Availability and implementation:**

The code and data are available on GitHub: https://github.com/Llannelongue/B4PPI.

## 1 Introduction

Protein–protein interactions (PPIs) are central to protein function and inform a wide range of biomedical applications, from mechanistic studies to drug development. Better understanding these interactions is critical for successfully mapping biological pathways, but the diversity of PPIs and the scale of the network make this a difficult task. Experimental methods to map PPIs exist, but, despite progress in systematic mapping, even high-throughput ones are not yet able to determine all possible interactions.

Through generalizable patterns learned on varied interaction data, computational methods can complement experiments by addressing the issue of scalability and measurement bias. Given a pair of proteins and some characteristics of each one, machine learning models can learn to predict the likelihood of interaction. Numerous methods have been developed for this, from early work on *S. cerevisiae* to algorithms dedicated to human PPIs. Yet, despite a wealth of tools, the mechanics and consequences of the underlying inference are still poorly understood, and it is unclear why models with similar performance make vastly different predictions. Reported performance scores often cannot be compared or replicated due to proprietary data and inconsistent or flawed assessment methods (Dunham and Ganapathiraju 2022, Bernett, Blumenthal and List 2023). This prompted recent efforts to benchmark published PPI prediction models more rigorously using common datasets and testing strategies (Dunham and Ganapathiraju 2022, Wang *et al.* 2023). These benchmarking studies hinted at some fundamental differences in how algorithms predict PPIs, e.g. some sequence-based models mostly leverage biases in the data (Dunham and Ganapathiraju 2022), which highlighted the need for inference mechanisms to be investigated further and with the same rigour.

In the absence of such studies, *in silico* PPI analyses are difficult to reconcile, the development of new models is inefficient, follow-up mechanisms studies are likely undermined and, ultimately, there are different versions of the underlying molecular networks that describe protein function. A range of publications have investigated best practices for machine learning in biology (Chicco 2017, Greener *et al.* 2022, Hou *et al.* 2022, Lee *et al.* 2022) and highlighted that replicable, trustworthy, and generalizable high-performing models can capture more causal biology and enhance many aspects of biological research such as experimental designs and drug development.

In this work, we study and compare two of the main building blocks of PPI prediction algorithms, based on functional genomic (FG) information or amino acid sequences alone. FG information includes gene ontology annotations, such as biological functions and cellular compartments, and gene expression profiles. We highlight why both perspectives are still relevant today and how each adapts to the PPI network’s topology. In particular, we show that the presence of highly connected proteins in the networks has a drastic impact on prediction models and is an area where FG and sequence models diverge. We also replicate these results between human and yeast (*S. cerevisiae*) and show how each tool performs on cross-species predictions. To elucidate the prediction mechanisms of these models, we design a robust and standardized approach to investigate *in silico* PPI prediction tools that accounts for both biological and statistical pitfalls and leverages the strength of large, open-source and professionally curated databases. We make publicly available benchmarking datasets for human and yeast PPIs to accelerate future discoveries and lay the foundations for similar datasets for other organisms. This work gives critical insight into each approach’s strengths and weaknesses and provides robust foundations for future developments in PPI prediction models.

## 2 Results

### 2.1 A robust and open-source benchmarking pipeline to understand PPI prediction models

**Figure 1.**
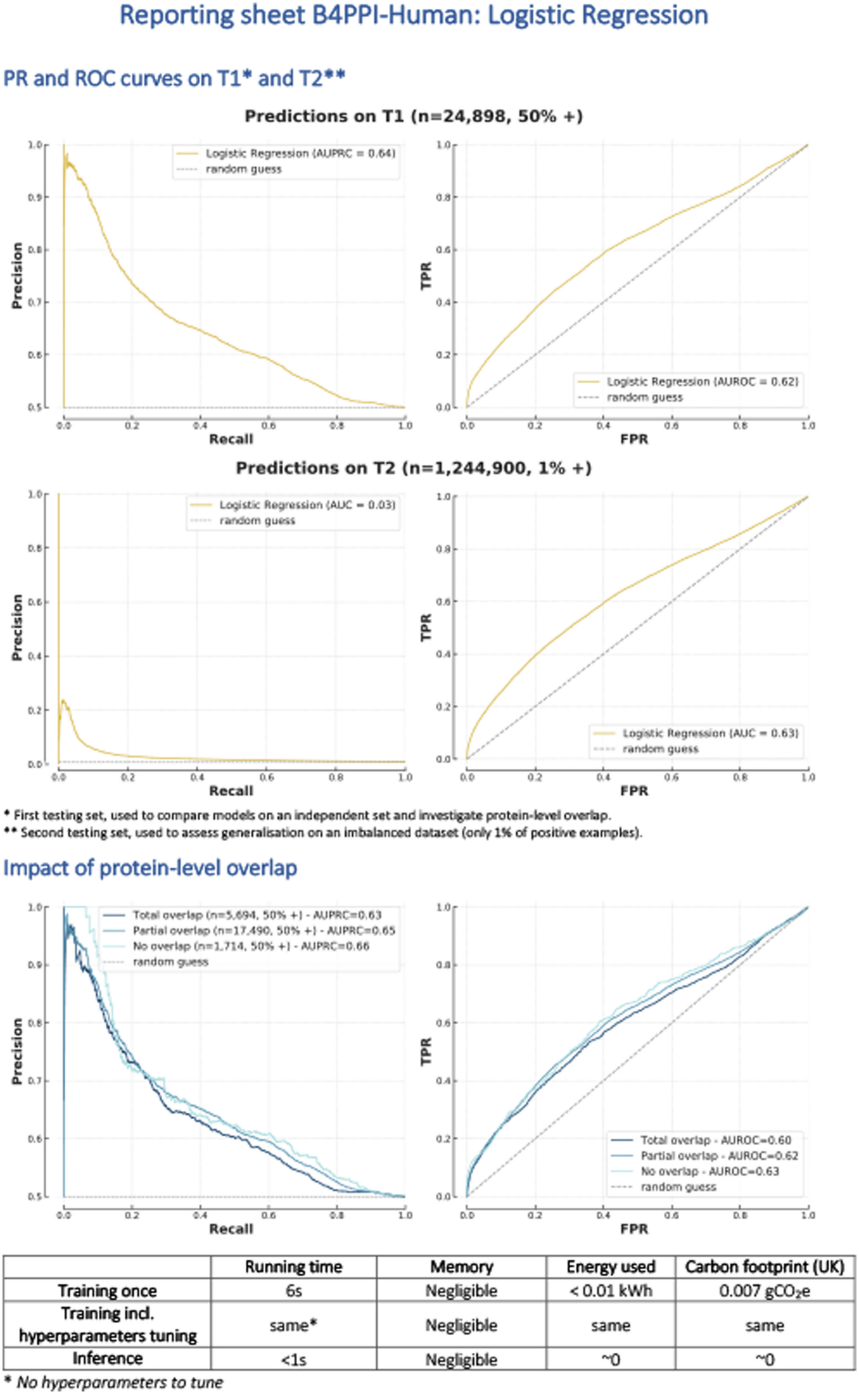
Reported performance sheet of the logistic regression (FG-based) on B4PPI-Human.

The lack of a consistent way to investigate PPI prediction algorithms has hindered their development and reduced their impact by making it difficult to reuse models for downstream analyses (Dunham and Ganapathiraju 2022). Benchmarks are important for replicability, and when combined with carefully curated datasets, they enable fast development through trial and error. Our Benchmarking Pipeline for the Prediction of Protein-Protein Interactions in Humans (B4PPI-Human) includes both carefully selected training and testing sets and a collection of input features to enable such trials. Standard UniProt IDs are used throughout to easily combine these with other data sources. Relevant metrics are selected with guidelines on how to share them. All this, alongside the pre-processing steps and relevant guidelines, is made available online and can be downloaded easily from https://github.com/Llannelongue/B4PPI. An example of a reporting sheet is in Fig. 1.

The complexity of the underlying biological mechanisms of PPIs introduces pitfalls that need to be considered when evaluating models. First, the way non-interacting proteins are selected for training is important. While some models have used proteins known to be localized in different parts of the cell, this has been shown to be unreliable and a source of significant bias that overestimates accuracy (Ben-Hur and Noble 2006). An alternative is to use a database of experimentally tested non-interacting proteins, but leading resources such as Negatome have only ∼1300 pairs and thus offer limited coverage. Considering the scarcity of PPIs, randomly sampling pairs of proteins has a very low risk of false negative and limits sampling bias (i.e. focusing on well-studied proteins) (Ben-Hur and Noble 2006, Park and Marcotte 2011). However, the impact of the associated imbalance between interacting and non-interacting proteins should be taken into account when training models on balanced datasets (Hu *et al.* 2021). Lastly, each observation is itself a pair of proteins. Even when ensuring that the two sets do not have pairs in common, there can be individual proteins present in both the training and testing sets. This protein-level overlap, often overseen, has been shown to significantly affect the performance of an algorithm and should therefore be properly assessed (Park and Marcotte 2012). Despite being documented in the literature, these pitfalls are still unevenly accounted for in published works (Dunham and Ganapathiraju 2022). This, alongside inconsistencies in the choice of testing sets and performance metrics explains why, despite the number of algorithms released in recent years, the underlying inference mechanisms are still not well understood.

The essential aspects of training and assessment that should be systematically accounted for are (i) the quality of the positive examples (i.e. the interacting proteins), (ii) how non-interacting proteins are selected for the gold standard, (iii) a suitable split between training and testing sets, in particular regarding individual proteins, and (iv) the metrics to evaluate and compare models. B4PPI seeks to address these four aspects of benchmarking.

When building a gold standard for machine learning algorithms, quality and representativity are the most important aspects to consider, which makes IntAct a database of choice for interacting proteins. It aggregates reliable evidence of molecular interactions from over 20 000 publications, which are manually curated, and includes data from other interactions databases such as the members of the IMEx consortium. Besides, by aggregating PPIs obtained from different experiments, each with different technical limitations, IntAct limits measurement bias. We further limited the risk of false positives by removing low-quality interactions, e.g. the ones based on spatial colocalization only (Supplementary Methods). The final dataset comprised 78 229 interactions, covering 12 026 proteins (out of the 20 386 registered in UniProt).

To select non-interacting proteins to serve as negative examples, randomly sampling protein pairs is the approach with the lowest probability of error considering the sparsity of the PPI network (Park and Marcotte 2011). Non-interacting proteins can be sampled using a uniform distribution, i.e.all proteins have an equal probability of being selected, which leads to an unbiased set, representative of the general population of protein pairs. However, PPI networks are known to be similar to scale-free networks, i.e. composed of a few highly connected nodes, called hubs, and numerous lone proteins with few interactions (Supplementary Fig. S1). Consequently, hubs are over-represented in a set of PPIs. For example in our curated set from IntAct, the top 20% of proteins by number of interactions were involved in 94% of PPIs. But when uniformly sampling protein pairs, the same top 20% were only involved in 37% of non-interacting proteins. Although expected, this can be an issue for machine learning algorithms that would identify hubs and systematically predict a positive interaction when hubs are involved. Such a strategy would maximize accuracy on the training set but lead to a majority of false positives when making predictions on new pairs. To mitigate this, a balanced sampling can be used (Yu *et al.* 2010), where the probability of sampling a protein for the negative set is proportionate to its frequency in the positive set. It has been shown that each strategy serves a different purpose (Park and Marcotte 2011); balanced sampling is beneficial for training models but should not be used for evaluating them. This was the strategy implemented here (Supplementary Methods).

In the presence of limited data, the division of the gold standard between training and testing sets is critical to simultaneously optimize learning and obtain meaningful generalization metrics. Here, the testing set should achieve several objectives, (i) provide performance metrics on a new, independent set, (ii) measure the impact, or absence of impact, of protein-level overlap, (iii) demonstrate how the model can generalize to real-world data. Since a single set cannot achieve simultaneously (ii) and (iii), as the careful selection of proteins to measure overlap biases the dataset, we designed two testing sets T1 and T2 (Supplementary Methods). T1 should be used to compare different approaches with an independent set and investigate protein-level overlap, and T2 should be used to assess generalization. T1 was built to include proteins purposefully left out of the training set; Supplementary Fig. S2 demonstrates the importance of this. T2, with only 1% of positive examples can then be used to assess how models perform in a more realistic setting where positive interactions are rare compared to non-interacting proteins.

The choice of metrics is a crucial element of a benchmark, and summarizing the results by a single number, such as accuracy or AUROC, is often misleading. We report both the ROC and the Precision-Recall (PR) curves which highlight nuanced and complementary aspects of PPI models. In addition, to address the environmental impacts of bioinformatics tools (Grealey *et al.* 2021), we also reported the carbon footprint of training models, measured using the Green Algorithms calculator (Lannelongue *et al.* 2021).

The elements described above represent the minimum needed for reproducible benchmarks and researchers who wish to use their own input features can evaluate their models on these partitions. However, to rapidly test a new model, it can be useful to have access to carefully curated protein properties. Amino acid sequences and functional genomics annotations, such as subcellular localization and biological functions are available with B4PPI, from the professionally curated databases UniProt, the Human Protein Atlas (HPA) and Bgee (Supplementary Methods for the full list of features). Supplementary Fig. S11 summarizes the benchmarking pipeline.

With B4PPI, we could compare different models in a consistent manner to better understand what aspects of the underlying biology are captured by each method. We focused here on FG-based and sequence-based models as they have been widely used and rarely compared, despite attempts at combining them.

### 2.2 FG-based linear models achieved top performance

In FG-based models, FG annotations are pre-processed to compute similarity measures, such as colocalization, between proteins (Supplementary Methods). The low dimensionality of the transformed problem explains the success of standard machine learning algorithms; in particular, Naïve Bayes Classifiers, decision trees and Random Forests have been the most popular choices (Jansen et al. 2003, Zhang *et al.* 2004, 2012, Chen and Liu 2005). Despite the proven track record of such tools, the more recent XGBoost algorithm has been shown to outperform them in other situations, which motivated its inclusion in this analysis.

We started with the simplest model, a logistic regression, and reported PR and ROC curves on the two test sets (Fig. 1). A list of coordinates for these curves was made available so that future models can be compared without unnecessary re-training. We also reported the training time, 6 s, and the carbon footprint, close to 0 gCO2e. We then tested other models and produced similar performance sheets (Supplementary Fig. S3).

**Figure 2.**
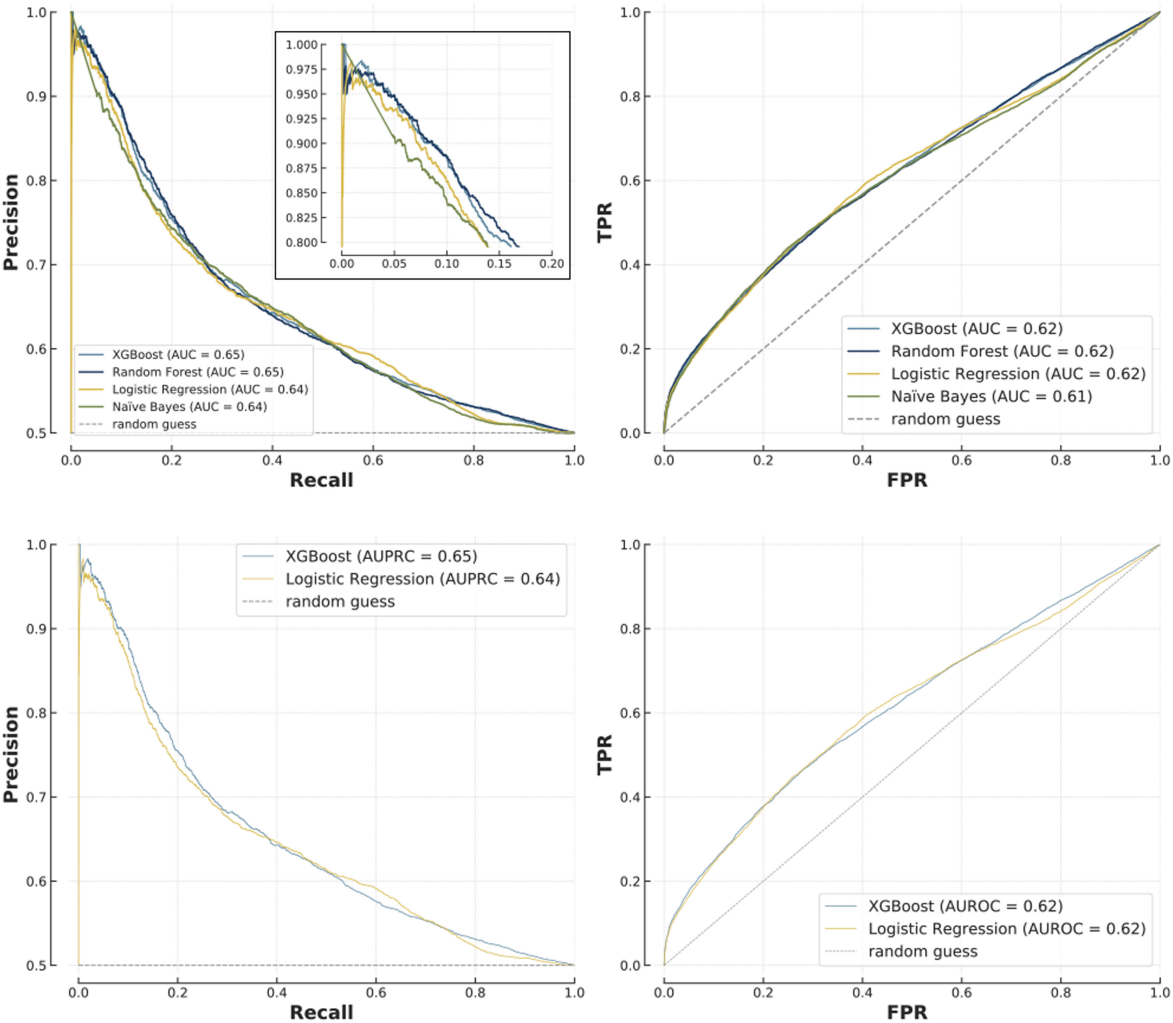
Comparison of FG-based models on *T1* (*n* = 24 898, 50% positive), with PR curves (left) and ROC curves (right) for all the models tested (top) and then only XGBoost and Logistic Regression for clarity (bottom). The top left plot also shows a zoom on the high precision/low recall area.

We found that more complex algorithms brought little improvement over logistic regression, as most models performed similarly on T1 (Fig. 2 and Supplementary Fig. S4). XGBoost and Random Forest showed minor improvement in AUROC and AUPRC, but the difference between the ROC curves of the logistic regression and XGBoost is non-significant (*P* = .27) (Supplementary Methods). Moreover, XGBoost was more efficient than Random Forest as it had nearly half the runtime (30 s versus 54 s). When studying the coefficients of the linear regression, we found that most decisions are based on common biological processes, co-localization (cellular compartment) and common domains, all three coefficients being significant (*P* < .001) (Supplementary Fig. S5). GO annotations for domains and motifs were missing for, respectively, 58% and 89% of proteins (Supplementary Methods), so we trained new FG-based models without them as a sanity check and found that it did not alter the results.

The reporting standard also enabled us to look at finer performance metrics, broken down by protein-level overlap (i.e. individual proteins common to the training and testing sets). Comparing PR and ROC curves showed that both logistic regression and XGBoost were unaffected by the level of overlap (Fig. 1 and Supplementary Fig. S3), and can therefore transfer effectively to new proteins.

### 2.3 Sequence models outperformed FG-based algorithms on known proteins

**Figure 3.**
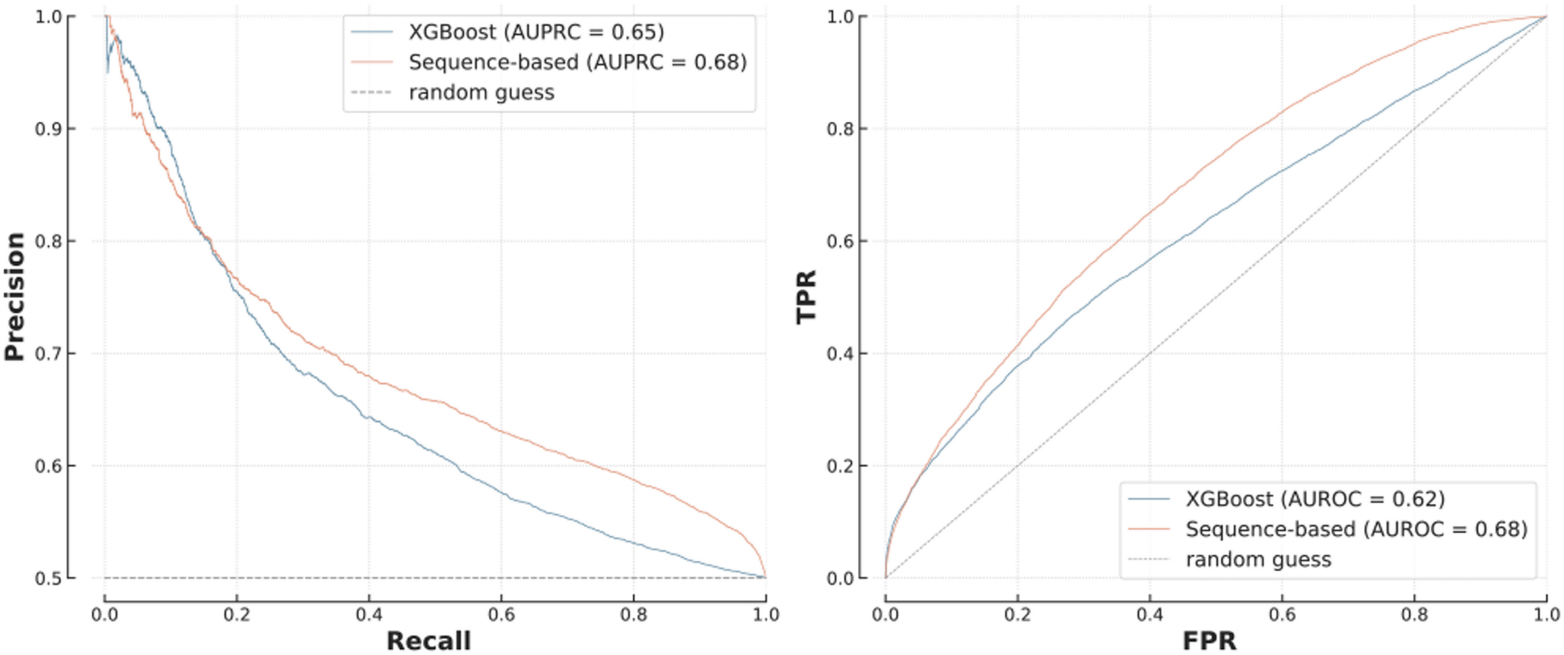
Siamese network versus XGBoost on *T1*. The difference between the ROC curves was statistically significant (*P* = 9 × 10^−47^).

An alternative to FG-based models is to use amino acids sequences as the input for a PPI prediction algorithm. We compared several deep learning architectures and reported the performance of an optimized Siamese recurrent neural network, used in other recent PPI models (Szymborski and Emad 2022) (Supplementary Methods and Supplementary Fig. S6). Despite not having access to functional information about the proteins, the sequence model outperformed the best performing FG-based model, XGBoost, except at low recall and high precision (AUPRC = 0.68 versus 0.65 and ROC curves significantly different, *P* = 9 × 10^−47^) (Fig. 3). However, while XGBoost was trained in only 30 s with less than 0.01 kWh of energy, the deep learning approach trained for 1h10 with 0.62 kWh, emitting 22 000 times more greenhouse gases (GHGs). In addition, the performance of the sequence model was heavily affected by the choice of deep learning architecture and its hyper-parameters, such as number of layers or learning rate. These require extensive (and expensive) optimization. We also confirmed that these results are not due to sequence homology between proteins in the training and testing sets, with the different models giving consistent results in different testing scenarios evaluating the impact of sequence similarity (Supplementary Methods and Supplementary Fig. S12).

Protein-level overlap had a significant impact on these results. The model had an AUPRC of 0.68 on average, but 0.75 when restricted to proteins present in both training and testing sets, and only 0.62 when there was no overlap (Supplementary Fig. S6). This demonstrates that (i) in the absence of specific adjustments, such deep learning models are poorly suited to make predictions on previously unseen proteins and (ii) in-depth benchmarks like B4PPI are important to reliably understand performance. While this comparison of FG-based and sequence-based models could indicate that deep learning is the best approach to PPI prediction, it could also be the consequence of unaccounted-for biological properties of PPIs.

### 2.4 The role of network hubs is essential to PPI prediction

**Figure 4.**
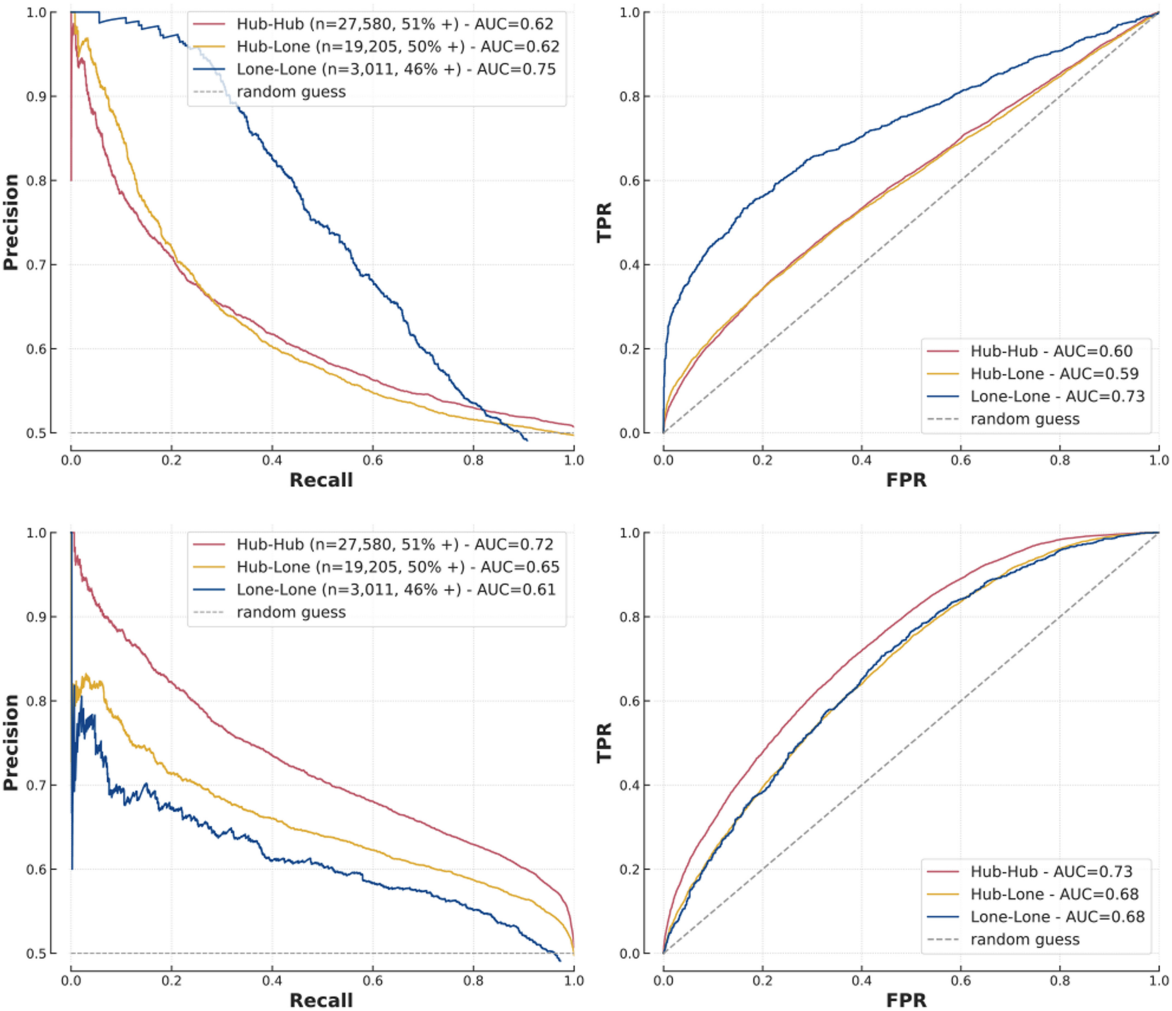
Performance of XGBoost (top) and sequence-based model (bottom) on hubs and lone proteins.

A scale-free topology has important biological implications so we hypothesized that a one-fits-all approach for hubs and lone proteins is unlikely to be optimal. Contrary to network-based prediction models which are, by design, expected to be particularly sensitive to topology, the impact on sequence-based and FG-based models is not so evident. In assessing interactions between protein hubs (hub-hub), between a protein hub and a lone protein (hub-lone) and between lone proteins (lone-lone) (Supplementary Methods), we found a distinct pattern whereby FG-based models had greater AUPRC and AUROC for interactions involving only lone proteins while sequence-based models performed better for hubs (Fig. 4).

These findings can be explained by the pre-processing of similarity measures for FG models. Because of their central role in biological pathways, hubs are highly studied and therefore annotated for many processes and localizations (more than other proteins, Supplementary Methods). Because the similarity measures used by the FG models quantify overlaps in annotations, hubs annotated for a large number of processes provide little information about the probability of interaction, which can explain why FG-based models perform best when hubs are not involved.

These results provide insight into the strengths of each approach and, importantly, show that a PPI approach should be context specific, particularly with respect to the network topologies of interest. Indeed, the apparent superiority of the deep learning model shown on Fig. 3 is largely due to the composition of T1, made up of 70% of hub–hub or hub–lone interactions.

### 2.5 Cross-species validation of PPI prediction models and relative performances

*Saccharomyces cerevisiae* is a well-studied model organism with a known interactome and has been used extensively for *in silico* PPI predictions (Ben-Hur and Noble 2005, Zhang *et al.* 2012). We replicated the analyses presented above on *S. cerevisiae* proteins and found that our findings regarding network topology and models’ relative performances were robust across species. The data were selected similarly to previously, extracted and curated from IntAct and UniProt, but without data from HPA and Bgee as these databases do not curate yeast (Supplementary Methods).

**Figure 5.**
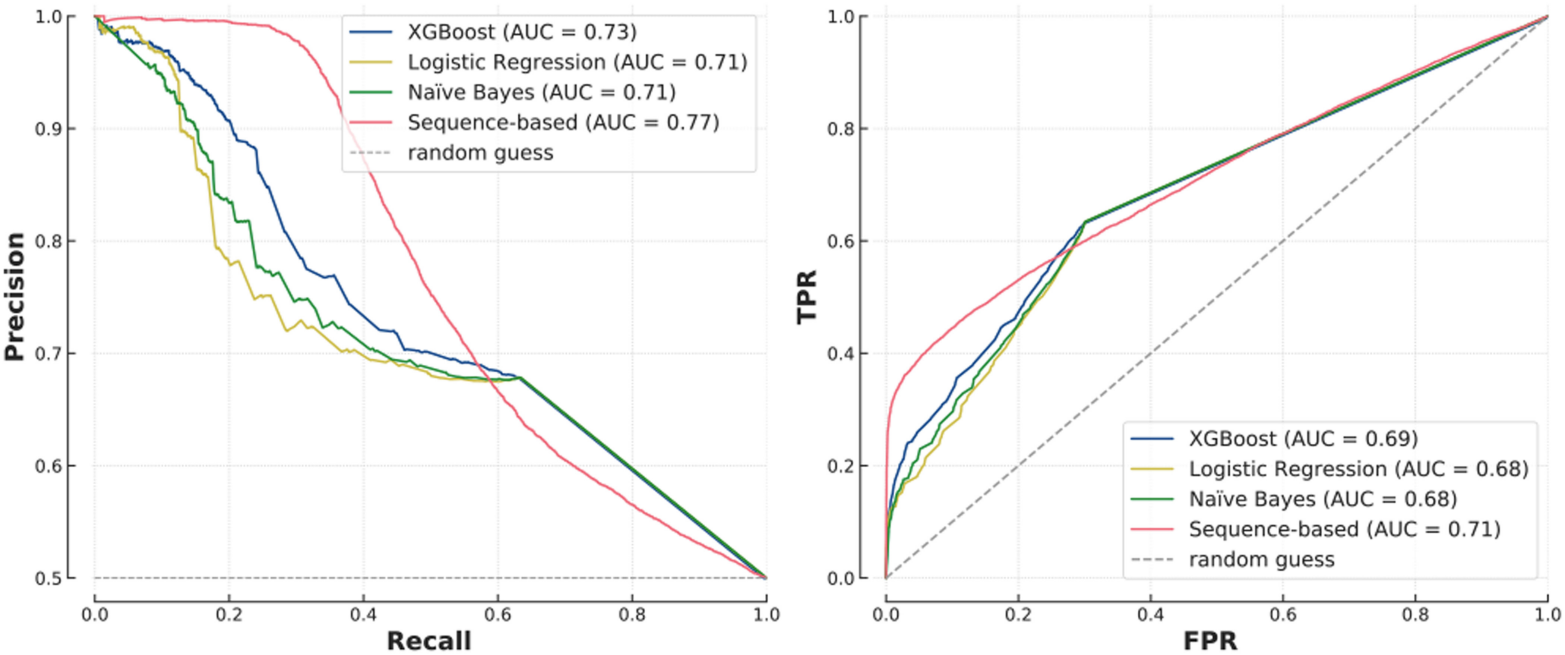
Comparison of a range of models on the yeast testing set.

As shown previously, all FG-based models had similar performances with AUPRC between 0.71 and 0.73 (Fig. 5); however, in this analysis, the differences between XGBoost and other models were statistically significant (*P* = 2 × 10^−12^ for Naïve Bayes and *P* = 9 × 10^−31^ for logistic regression). The sequence model outperformed FG-based models in most cases (*P* = 2 × 10^−6^), except at high recall (Fig. 5). Secondly, similar to human proteins, FG-based models were not sensitive to protein-level overlap while sequence-based models had different performances depending on the level of overlap (Supplementary Fig. S7). Finally, we found consistency regarding the role of network hubs; FG-based models were better able to predict lone–lone protein interactions while the sequence-based model was better at predicting interactions amongst protein hubs (Supplementary Fig. S8).

While experimental data on PPIs is readily available for humans and *S. cerevisiae*, many non-model organisms lack data despite their biological relevance. For these, cross-species predictions—i.e. training a model on a species to make predictions on another—are of particular interest. We showed that FG-based models are generally more suitable than sequence-based ones for this task.

**Figure 6.**
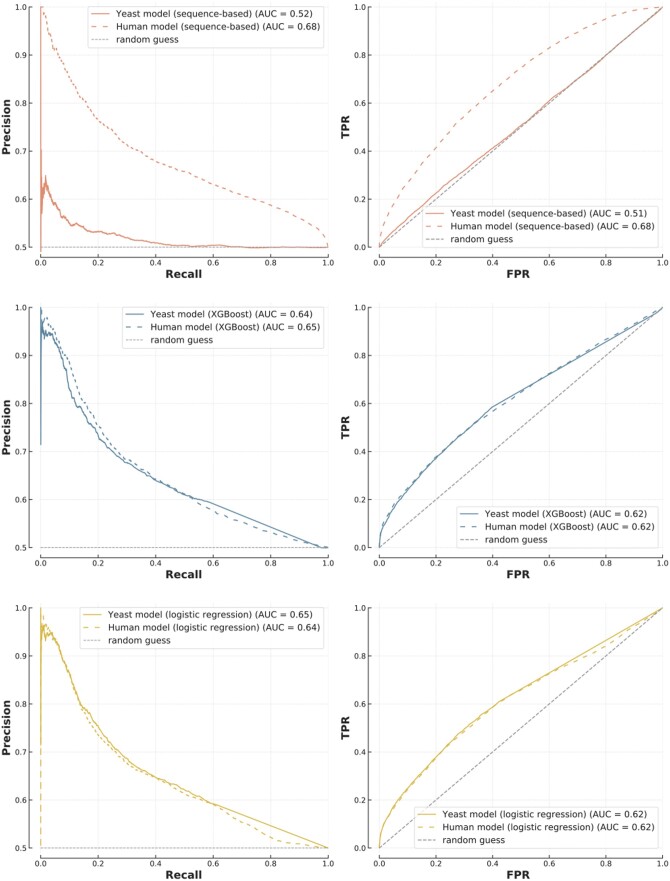
Cross-species predictions. Models trained on human PPIs (dotted lines) and yeast PPIs (solid lines) were used to make predictions on the human testing set. The top plot is the sequence-based model, the other ones are FG-based (XGBoost in the middle, logistic regression at the bottom)

We investigated whether models trained on yeast could be used to predict human PPIs, finding that the yeast-trained FG-based models (logistic regression and XGBoost) achieved similar AUPRC and AUROC as those which were human-trained to predict human PPIs (*P* = .26 for XGBoost) (Fig. 6). Conversely, the yeast-trained sequence model was unable to predict human PPIs (AUPRC = 0.52 versus 0.68, *P* = 3 × 10^−272^). We observed the same phenomenon when using human-trained models to make predictions on yeast (Supplementary Fig. S9).

## 3 Discussion

In this work, we sought to identify and explain the strengths and weaknesses of PPI prediction approaches, and thereby provide the community with both a benchmarking pipeline and insight into which PPI approach to select or trust in a particular scenario.

The FG-based and sequence-based models are commonly used as building blocks for PPI prediction but are rarely directly compared. In particular, it was unclear where the differences lie and if one approach should be preferred today. The success of FG-based models, which use gene ontologies among other variables, is in line with previous results (Armean *et al.* 2018), and we found that when using FG annotations, the choice of algorithm has little impact on the predictions and a logistic regression performs close to the state-of-the-art while providing clear insight into the decision-making process (Fig. 2). Here, colocalization, common biological processes and shared domains are the main indicators of interaction (Supplementary Fig. S5). Interestingly, different publications tend to highlight different FG features depending on the organism, dataset and predictor (mRNA expression in Zhang *et al.* (2004), coexpression/colocalization in Srinivasan *et al.* (2006) or biological process in Armean *et al.* (2018)). The fact that a highly flexible and non-linear model such as XGBoost performs similarly to logistic regression, making identical predictions in 93% of cases, shows that performance is likely driven by the quality and the pre-processing of the FG annotations instead of the modelling; once the similarity measures have been calculated, there are limited non-linearities and a simple logistic regression achieves top performance. Sequence-based models on the other hand need specifically optimized architectures but achieve similarly high performances, if not higher in some settings, without any biological information apart from amino acid sequences (Fig. 3).

We found that the two approaches adapt to the presence of hubs and lone proteins differently and show complementary strengths. While sequence-based models are mostly useful when hubs are involved, FG-based models perform well for interactions between lone proteins (Fig. 4). This simple result offers important insight into the specificities of each approach and explains discrepancies in reported performances in the literature, as the topology of the testing set has a large impact on metrics. These results are not specific to human PPIs as the same conclusions were drawn from analysis on S. cerevisiae (Supplementary Fig. S8). Cross-species predictions are instrumental to study non-model organisms, and we showed that FG-based decision rules translate well to new species while sequence-based models do not.

These observations are consistent with the way each algorithm learns. FG-based models make predictions based on general, but less complex, rules about PPIs which translate well to new proteins and new species. This is particularly useful considering that many proteins are still not represented in interaction databases. On the other hand, sequence-based models have millions of parameters which give them the flexibility to recognize individual proteins and learn specific interaction patterns. Although this enables such models to make predictions without functional information, it also limits high performance to proteins present in the training set, and make them particularly susceptible to data leakage (Bernett *et al.* 2023). This likely explains the poor results of sequence models on previously unseen proteins and cross-species datasets (Fig. 6), something also observed by Dunham *et al.* in their benchmarking effort (Dunham and Ganapathiraju 2022). It is also consistent with the high performance of these models on network hubs, which are overrepresented in training sets and therefore well captured by the models.

These analysis and results required a robust and reliable benchmarking pipeline. We designed the open-source B4PPI (Supplementary Fig. S11), which accounts for a range of biological and statistical pitfalls. By being freely accessible and using standard identifiers for proteins, B4PPI can be used by any researcher working on in silico PPI prediction to investigate the inference mechanisms of their models. While ROC and PR are the two performance scores considered in this study, any metric can be used and included in the pipeline. Contrary to previous benchmarking efforts (Wang *et al.* 2023), which it can complement, B4PPI leverages large professionally curated databases. An example reporting sheet is presented (Fig. 1) that includes relevant metrics to ensure the models released can be trusted and encourage wider use of PPI imputation for downstream analysis. Such templates are not yet common in benchmarks, but would promote more exhaustive and granular reporting of models’ performances. B4PPI also comes with pre-processed features to enable rapid development of new approaches.

Our study has limitations. We focused on two widely used approaches to PPI prediction, namely FG-based and sequence-based; however, some alternative approaches have also been proposed, using, for example, higher-level protein structures (Zhang *et al.* 2012), phylogeny (Marmier *et al.* 2019), and networks topology (Wang *et al.* 2023). Most FG annotations are from gene ontologies which have a hierarchical structure which we do not account for here, contrary to Armean *et al.* (2018) for example. We also do not address the dynamic nature of PPIs in this work, and while the methods discussed here provide likelihood of interactions, these can be further impacted by spatiotemporal and cell-specific regulation mechanisms. Moreover, we analysed two common interactomes, human and yeast, yet there are many more. As demonstrated though with the yeast dataset, similar benchmarks and analysis can be transferred to other model organisms in a relatively straightforward manner. Literature-based databases such as IntAct are known to suffer from sampling bias as the curated studies tend to focus on already well-researched proteins (Rolland *et al.* 2014), which may skew the predictions. One strategy to avoid sampling bias is to include high-throughput systematic mapping experiments, and IntAct—and therefore B4PPI—integrates a number of those, such as HuRI and BioPlex.

We showed here the limits of classic sequence-based deep learning models on new proteins and for cross-species predictions, but it is worth noting some recent deep learning models that potentially addressed these limitations through careful regularization for the former (Szymborski and Emad 2022) and by including biological and chemical information about amino acids as well as structural knowledge for the latter (Mahapatra and Sahu 2021, Sledzieski *et al.* 2021). The results presented in this work can hopefully guide similar future work and help move this area further.

While benchmarking standards for PPI prediction are needed, it is important to remember the downsides of benchmarks, as demonstrated in computer vision or natural language processing. A fixed set of metrics can motivate the community to overly focus on those, at the expense of applicability and usefulness. To limit this, B4PPI includes a range of metrics but the relevant indicators for each use-case should nonetheless be carefully considered.

The size and complexity of the PPI network makes in silico prediction tools indispensable, but it is important to ensure that the models developed are reliable and readily available to the community for downstream analysis and to give insights into biological pathways. For this, consistent and reliable evaluation pipelines are necessary as well as a better understanding of what machine learning models learn. The results presented here make key progress in both areas and facilitate the development, evaluation and reliability of future PPI models.

## Supplementary Material

btae012_Supplementary_Data
