# Supplementary material for: Pitfalls of machine learning models for protein–protein interaction networks
Source: Bioinformatics. 2024 Jan 10;40(2):btae012. doi: 10.1093/bioinformatics/btae012 (PMC10868344; doi:10.1093/bioinformatics/btae012)
Supplement: btae012_Supplementary_Data [file btae012_supplementary_data.pdf]

SUPPLEMENTARY METHODS

B4PPI-Human

The data was obtained from large and professionally curated databases. This limits measurement bias, as each interaction is based on several experiments, and leverages experts' knowledge in the curation process. On average, each PPI is supported by 3.4 publications (median of 3) and only 4.9% of interactions are obtained from only one source. Standard UniProt IDs are used throughout to ensure maximum compatibilities. **Supplementary Figure 11** summarises the benchmarking pipeline described below.

Most of the manipulations were done in Python (The Python Language Reference — Python 3.10.1 documentation) with Jupyter Notebooks (Jupyter Project Documentation — Jupyter Documentation 4.1.1 alpha documentation) using the Pandas library (McKinney 2010; Reback *et al.* 2020) and Numpy (Harris *et al.* 2020). The plots were drawn using Matplotlib (Hunter 2007), Seaborn (Waskom 2021) and the MetBrewer colour palettes (GitHub - BlakeRMills/MetBrewer: Color palette package in R inspired by works at the Metropolitan Museum of Art in New York). All the code and final data are available on GitHub (https://github.com/Llannelongue/B4PPI); some intermediary pre-processed datasets are not available online due to file size limits but they can be recreated using the code available. Data is available under Creative Commons Attribution (CC BY 4.0) License.

Protein-protein interaction data

To train machine learning algorithms, the quality of the gold standard is paramount. Data on PPIs was obtained from IntAct (Orchard *et al.* 2014) and downloaded from the EMBLE-EBI FTP server (timestamp: 15/10/2021). We restricted the data to human heterodimeric protein-protein interactions with UniProt IDs. To reduce the risk of false positives, we removed complex expansions (where the pairwise interactions within a complex are unreliable) and interactions based on colocalisation only. This quality control step leaves 128,790 PPIs, covering 15,506 proteins (out of 20,386 in UniProt). Based on this dataset, we created an index of the number of recorded interactions per protein and made a list of hubs (highly connected proteins). In line with the literature, hubs are defined as the 20% of proteins with the most interactions (Jin *et al.* 2007), which here is equivalent to proteins with more than 21 partners. The quality of the interactions is assessed further by looking at the MIscore (IntAct - User Guide), a quality score based on the manual curation of the interactions and annotations of the HUPO PSI-MI consortium that takes into account the detection method, the interaction type and the number of publications reporting it. In case of PPIs with multiple entries, the highest MIscore was used. When looking at the distribution of the MIscores in the dataset (**Figure 1**), a threshold of 0.47 is visible, which restrict the dataset to 78,229 interactions, covering 12,026 proteins. We also ran the same analyses without filtering on MIscores (i.e. using all 128,790 interactions) and found that all the results presented here held true.

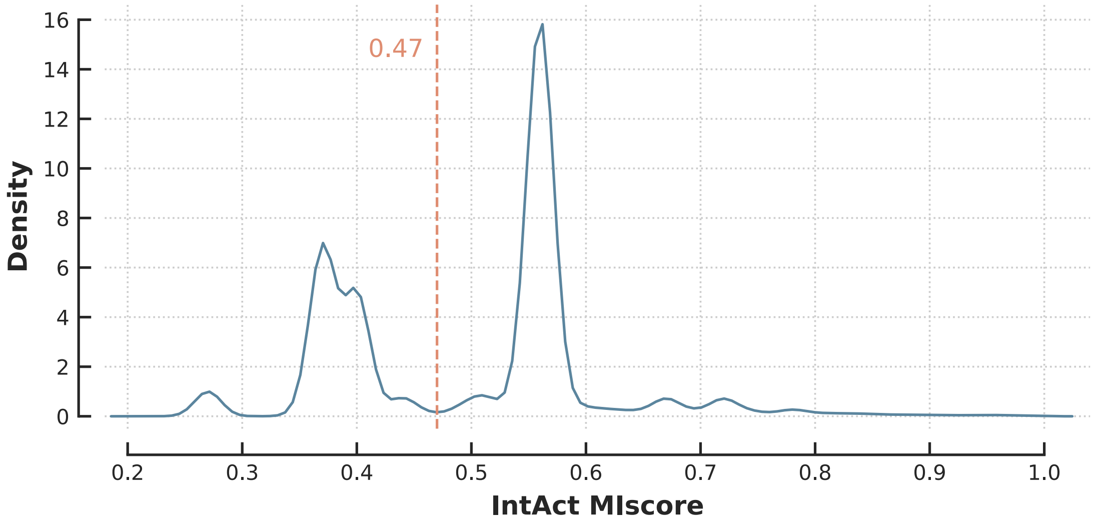

Figure 1: Distribution of the MIscore in IntAct.

Functional genomics annotations and amino acids sequences

Protein sequences in humans are well documented and can be obtained from UniProt, but FG features can be more challenging as they should be diverse (i.e. cover a wide range of properties), of high-quality and have high coverage (i.e. few missing proteins). For the same reasons as described above, aggregated, manually curated, and professionally reviewed databases are preferred. Based on features that have been successfully used for the task before, it is relevant to include information about cellular and tissue localisation, biological functions and gene expression patterns (Jansen 2003; Ben-Hur and Noble 2005; Zhang *et al.* 2012; Kotlyar *et al.* 2015).

One of the main databases on proteins is UniProt (The UniProt Consortium 2021) and in particular its knowledgebase UniProtKB. Swiss-Prot, the section of UniProtKB that is reviewed and manually curated, is used in this work to ensure optimal quality. The data from Swiss-Prot is downloaded through their API by restricting to reviewed, non-obsolete, human proteins (last download is 09/11/2021). The different columns are then cleaned to extract the information of interest in a standardised format, and we use UniProt IDs throughout. There is information for 20,386 proteins and more details about each feature are in **Table 1**. UniProt's API is also used to map UniProt IDs between different databases and to map outdated IDs. In particular, we extract amino acids sequences for each protein, more than 95% of which come from the translation of coding sequences submitted to the International Nucleotide Sequence Database Collaboration (Arita *et al.* 2021). Annotated domains and motifs are also included in the database. Additionally, we extract gene ontology (GO) annotations of biological processes, cellular components and molecular functions. For each protein, each of the FG features is represented as a bag-of-words, i.e. a sparse vector of length the number of annotations in the database.

When working with gene expression data, both biological and technical noise need to be accounted for correctly. The Bgee public repository (Bastian *et al.* 2021) does that by regrouping curated healthy wild-type standardised gene expression patterns. The human data is mainly from GTEx v6 (phs000424.v6.p1), with an added layer of manual curation to remove unhealthy subjects. For a gene, the final data provides binary calls of presence or absence of expression for each

combination of anatomical entity and developmental stage. We downloaded the database from their FTP server (version 14.2) and obtained information for 59,777 genes, 320 anatomical entities and 33 developmental stages, which leads to 1,147 stage/entity combinations. The Bgee entries are matched to the UniProt IDs using UniProt's own mapping table.

The Human Protein Atlas (HPA) (Uhlén *et al.* 2015; Thul *et al.* 2017) provides data mapping human proteins to tissues and cells. In particular, we used the Tissue Atlas (Uhlén *et al.* 2015) that presents the distribution of proteins in tissues and cell types and the Cell Atlas (Thul *et al.* 2017) that contains the distribution across subcellular locations. The Tissue Atlas contains data similar to Bgee, but the overlap is likely to be limited as the two databases only share GTEx RNA-seq data. While Bgee has a more thorough curation process, HPA contains a lot of original in-house experimental results, which justifies the inclusion of both data sources. We downloaded the HPA data from their website (release 20.1, Ensembl version 92.38). The data in HPA is identified by Ensembl gene IDs, which are mapped to UniProt IDs using UniProt's API. We restricted the dataset to the reviewed proteins present in Swiss-Prot and to ensure the quality of annotations, we discarded the entries HPA annotated as "uncertain". For the tissue IHC data, we mapped expression levels to numerical values (high=3, medium=2, low=1 and "not detected"=-1) with untested tissues being mapped to 0. Similar pre-processing was used for the consensus RNA-seq data and the subcellular location.

Table 1: Features used to train human models (GO = Gene Ontology).

Feature	Number of different annotations	Missing values (/20,386)	Source
Biological processes (GO)	12,248	3,338	UniProt (The UniProt Consortium 2021)
Cellular components (GO)	1,754	1,765	UniProt
Molecular functions (GO)	4,346	4,552	UniProt
Domains	2,313	11,815	UniProt
Motifs	819	18,103	UniProt
Sequence	N/A	0	UniProt
Gene expression profile	1,147	1,296	Bgee (Bastian *et al.* 2021)
Tissue IHC data	62	9,536	HPA (Uhlén *et al.* 2015; Thul *et al.* 2017)
Tissue and cell type	189	9,536	HPA
RNA-seq	61	1,448	HPA
Subcellular location	33	7,820	HPA

Pre-processing to measure features similarity

For a protein, each FG feature was represented as a vector, of length the number of annotations. To measure the feature-specific similarity between two proteins, we compared the two vectors using cosine similarity(Singhal 2001), a popular tool widely used for similar tasks in Natural Language Processing. For two vectors $A = (A_i)$ and $B = (B_i)$, their cosine similarity $CS(A, B)$ is:

$$CS(A,B) = \frac{A \cdot B}{\|A\| \, \|B\|} = \frac{\sum_i A_i B_i}{\sqrt{\sum_i A_i^2} \sqrt{\sum_i B_i^2}}$$

As a result, for each of the 207,784,305 possible pairs of proteins, we obtained 12 similarity features: biological processes, cell components, molecular function, domains and motifs from UniProt, gene expression from Bgee, tissue/cell expression, tissue expression, RNA-seq expression and subcellular locations from the Human Protein Atlas (**Table 1**).

Creation of the gold standard

The PPIs obtained from IntAct are divided between a training set and two testing sets. First, a set of 1,562 proteins (13%) was randomly set aside to ensure some unseen proteins are present in the testing set; the necessity of this is shown in **Supplementary Figure 2**. This percentage was chosen as it gives a train/test ratio of ~70%/30%, which is standard when data is limited. The dataset was then randomly divided under this constraint and included 53,331 PPIs in the training set, 12,449 in *T1* and the same in *T2* (**Supplementary Table 1**). We tested the sensitivity of the results to the division parameters by creating new gold standards where 10% and 15% of proteins were set aside for testing (instead of the 13% mentioned above) and found that all the results remained identical.

The negative examples (i.e. non interacting proteins) are obtained using random sampling among all the possible pairs, excluding any pair that has been observed experimentally to limit the risk of false negative. For the training set, balanced sampling is used (Yu *et al.* 2010) to favour learning, which means that the probability of sampling a protein for the negative set is proportionate to its frequency in the positive set. For *T1* and *T2*, we used uniform sampling (all proteins have the same probability of sampling) to limit sampling bias. The training set and *T1* both have 50% of positive examples, while *T2* has only 1% of interacting pairs (**Supplementary Table 1**).

To confirm that the results obtained are not due to sequence homology between the training and testing sets, we designed two additional testing scenarios. For the first one, following a commonly used rule(Greener *et al.* 2022; Hou *et al.* 2022), we limited sequence similarity between any distinct training and testing proteins to 25%. The second scenario was more restrictive and excluded all paralogs of training proteins from the testing set. **Supplementary Figure 12** shows a comparison of the different model's performance in these different scenarios, and confirms that the results reported are not due to sequence homology.

To investigate how models deal with different network topologies, especially hubs and lone proteins, we had to create a separate testing set to ensure sufficient sample size in each category (hub-hub, hub-lone and lone-lone interactions). We do so by aggregating PPIs from *T1* and *T2*, and using balanced sampling for the non-interacting proteins. This results in 49,796 pairs (50% positive) (**Supplementary Table 2**).

We quantified the difference in FG annotations between hubs and non-hubs. For example in the training set, hubs have on average 11.6 annotations for biological processes (significant feature in the logistic regression model discussed in **Results**) while non-hubs only have 5.8 (median 6 vs 3). The same phenomenon is observed for cellular compartments (6.2 vs 3.6 annotations on average).

S. cerevisiae data

The pipeline describe above was also followed for the *S. cerevisiae* data. UniProt lists 6,721 yeast proteins and the same information as for humans (**Supplementary Table 3**) but HPA and Bgee do not include data for this organism. PPIs were obtained from IntAct following the same procedure, although no selection based on MIscores was made considering the absence of an obvious choice when looking at the distribution (**Supplementary Figure 10**). The final PPI dataset comprised 43,068 interactions covering 5,679 proteins.

The split between training and testing sets was done similarly by setting aside 737 proteins for testing and then randomly allocating PPIs to keep 30,369 PPIs for training (70% of the gold standard). Because there is fewer data on yeast, and only one testing set is needed to replicate the analysis conducted on humans, dividing the remaining 12,699 further between *T1* and *T2* is not suitable here. But if the goal was to measure generalisability of a yeast model, this could be easily done.

Training

FG-based machine learning models were trained using the scikit-learn library (Pedregosa *et al.* 2011). For models that cannot deal with missing data, mean imputation was used (**Supplementary Table 4**). Hyper-parameter search was done using Weight-and-Bias's Bayesian method (Biewald 2020) to find the optimal settings of each algorithm in a reasonable time. All hyperparameter choices are in **Supplementary Table 4** and **Supplementary Table 5**.

Deep learning models were trained using PyTorch Lightning (Falcon and The PyTorch Lightning team 2019; Paszke *et al.* 2019). The Siamese architecture (Bromley *et al.* 1994; Chopra, Hadsell and LeCun 2005) was composed of a bidirectional Gated Recurrent Unit (GRU) (Cho *et al.* 2014) followed by a linear output (**Figure 2**). Long-Short Term Memory networks (LSTM) (Hochreiter and Schmidhuber 1997) and Convolutional Neural Networks (CNN) (LeCun *et al.* 1990) were also tested, but GRU was preferred because of runtime efficiency and its ability to account for proteins of various lengths. Full parameters are in **Supplementary Table 4**, **Supplementary Table 5** and in the open-source code.

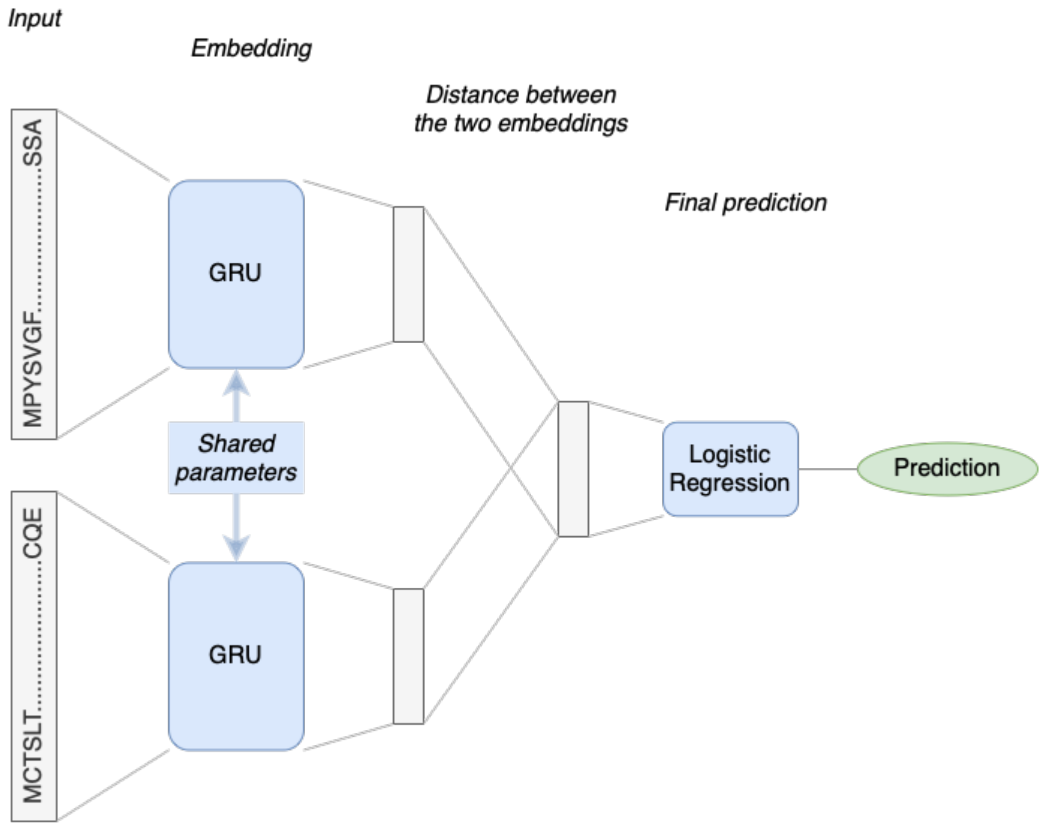

Figure 2: Diagram of the deep learning architecture used to predict interactions from a pair of protein sequences.

Evaluation

The Receiver Operating Characteristic (ROC) and the Precision-Recall (PR) curves are complementary options for PPI prediction. While the ROC curve is unaffected by the prevalence of interacting proteins, a benefit as the true prevalence of PPIs is mostly unknown, it also means that both classes are considered equally, whereas often, PPIs are more interesting than non-interacting proteins. This is addressed by the PR curve where precision puts an emphasis on positive examples. It has also been shown that ROC tend to overestimate the performance of PPI prediction tools (Wang *et al.* 2023), but most published models still report it, which justifies the inclusion of both metrics.

Both curves are reported alongside their respective Areas Under the Curve (AUC). To statistically compare ROC curves for a same testing set, we used a DeLong nonparametric test(DeLong, DeLong and Clarke-Pearson 1988) and reported the p-value. We corrected for multiple testing by using a conservative significance threshold of 5×10^{-4}, corresponding to a Bonferroni correction for 100 pairwise comparisons(Neyman and Pearson 1928).

Environmental impact statement

We did our best to minimise greenhouse gas emissions related to this project and, using the Green Algorithms calculator (v2.1)(Lannelongue, Grealey and Inouye 2021), we estimated that the carbon footprint of this project was 51 $kgCO_2e$, which corresponds to 4.7 tree-years.

References

Arita M, Karsch-Mizrachi I, Cochrane G *et al.* The international nucleotide sequence database collaboration. *Nucleic Acids Research* 2021;**49**:D121–4.

Bastian FB, Roux J, Niknejad A *et al.* The Bgee suite: integrated curated expression atlas and comparative transcriptomics in animals. *Nucleic Acids Research* 2021;**49**:D831–47.

Ben-Hur A, Noble WS. Kernel methods for predicting protein-protein interactions. *Bioinformatics* 2005;**21**:i38–46.

Biewald L. Experiment Tracking with Weights and Biases. 2020.

Bromley J, Guyon I, LeCun Y *et al.* Signature Verification using a "Siamese" Time Delay Neural Network. In: Cowan JD, Tesauro G, Alspector J (eds.). *Advances in Neural Information Processing Systems 6*. Morgan-Kaufmann, 1994, 737–44.

Cho K, van Merrienboer B, Gulcehre C *et al.* Learning Phrase Representations using RNN Encoder-Decoder for Statistical Machine Translation. *arXiv:14061078 [cs, stat]* 2014.

Chopra S, Hadsell R, LeCun Y. Learning a Similarity Metric Discriminatively, with Application to Face Verification. *2005 IEEE Computer Society Conference on Computer Vision and Pattern Recognition (CVPR'05)*. Vol 1. San Diego, CA, USA: IEEE, 2005, 539–46.

DeLong ER, DeLong DM, Clarke-Pearson DL. Comparing the Areas under Two or More Correlated Receiver Operating Characteristic Curves: A Nonparametric Approach. *Biometrics* 1988;**44**:837.

Falcon W, The PyTorch Lightning team. PyTorch Lightning. 2019, DOI: 10.5281/zenodo.3828935.

GitHub - BlakeRMills/MetBrewer: Color palette package in R inspired by works at the Metropolitan Museum of Art in New York.

Greener JG, Kandathil SM, Moffat L *et al.* A guide to machine learning for biologists. *Nat Rev Mol Cell Biol* 2022;**23**:40–55.

Harris CR, Millman KJ, van der Walt SJ *et al.* Array programming with NumPy. *Nature* 2020;**585**:357–62.

Hochreiter S, Schmidhuber J. Long Short-Term Memory. *Neural Computation* 1997;**9**:1735–80.

Hou Q, Waury K, Gogishvili D *et al.* Ten quick tips for sequence-based prediction of protein properties using machine learning. Palagi PM (ed.). *PLoS Comput Biol* 2022;**18**:e1010669.

Hunter JD. Matplotlib: A 2D Graphics Environment. *Comput Sci Eng* 2007;**9**:90–5.

IntAct - User Guide.

Jansen R. A Bayesian Networks Approach for Predicting Protein-Protein Interactions from Genomic Data. *Science* 2003;**302**:449–53.

Jin G, Zhang S, Zhang X-S *et al.* Hubs with Network Motifs Organize Modularity Dynamically in the Protein-Protein Interaction Network of Yeast. Monk N (ed.). *PLoS ONE* 2007;**2**:e1207.

Jupyter Project Documentation — Jupyter Documentation 4.1.1 alpha documentation.

Kotlyar M, Pastrello C, Pivetta F *et al.* *In silico* prediction of physical protein interactions and characterization of interactome orphans. *Nature Methods* 2015;**12**:79–84.

Lannelongue L, Grealey J, Inouye M. Green Algorithms: Quantifying the Carbon Footprint of Computation. *Advanced Science* 2021;**8**:2100707.

LeCun Y, Boser B, Denker J *et al.* Handwritten Digit Recognition with a Back-Propagation Network. *Advances in Neural Information Processing Systems*. Vol 2. Morgan-Kaufmann, 1990.

McKinney W. Data Structures for Statistical Computing in Python. Austin, Texas, 2010, 56–61.

Neyman J, Pearson ES. On the Use and Interpretation of Certain Test Criteria for Purposes of Statistical Inference: Part I. *Biometrika* 1928;**20A**:175–240.

Orchard S, Ammari M, Aranda B *et al.* The MIntAct project--IntAct as a common curation platform for 11 molecular interaction databases. *Nucleic Acids Res* 2014;**42**:D358-63.

Park Y, Marcotte EM. Flaws in evaluation schemes for pair-input computational predictions. *Nature Methods* 2012;**9**:1134–6.

Paszke A, Gross S, Massa F *et al.* PyTorch: An Imperative Style, High-Performance Deep Learning Library. *Advances in Neural Information Processing Systems*. Vol 32. Curran Associates, Inc., 2019.

Pedregosa F, Varoquaux G, Gramfort A *et al.* Scikit-learn: Machine Learning in Python. *J Mach Learn Res* 2011;**12**:2825–30.

Reback J, McKinney W, jbrockmendel *et al.* pandas-dev/pandas: Pandas 1.0.3. 2020, DOI: 10.5281/zenodo.3715232.

Singhal A. Modern Information Retrieval: A Brief Overview. 2001:9.

The Python Language Reference — Python 3.10.1 documentation.

The UniProt Consortium. UniProt: the universal protein knowledgebase in 2021. *Nucleic Acids Research* 2021;**49**:D480–9.

Thul PJ, Åkesson L, Wiking M *et al.* A subcellular map of the human proteome. *Science* 2017;**356**, DOI: 10.1126/science.aal3321.

Uhlén M, Fagerberg L, Hallström BM *et al.* Tissue-based map of the human proteome. *Science* 2015;**347**, DOI: 10.1126/science.1260419.

Wang X-W, Madeddu L, Spirohn K *et al.* Assessment of community efforts to advance network-based prediction of protein–protein interactions. *Nat Commun* 2023;**14**:1582.

Waskom M. seaborn: statistical data visualization. *JOSS* 2021;**6**:3021.

Yu J, Guo M, Needham CJ *et al.* Simple sequence-based kernels do not predict protein-protein interactions. *Bioinformatics* 2010;**26**:2610–4.

Zhang QC, Petrey D, Deng L *et al.* Structure-based prediction of protein–protein interactions on a genome-wide scale. *Nature* 2012;**490**:556–60.

SUPPLEMENTARY MATERIAL

Supplementary Figures

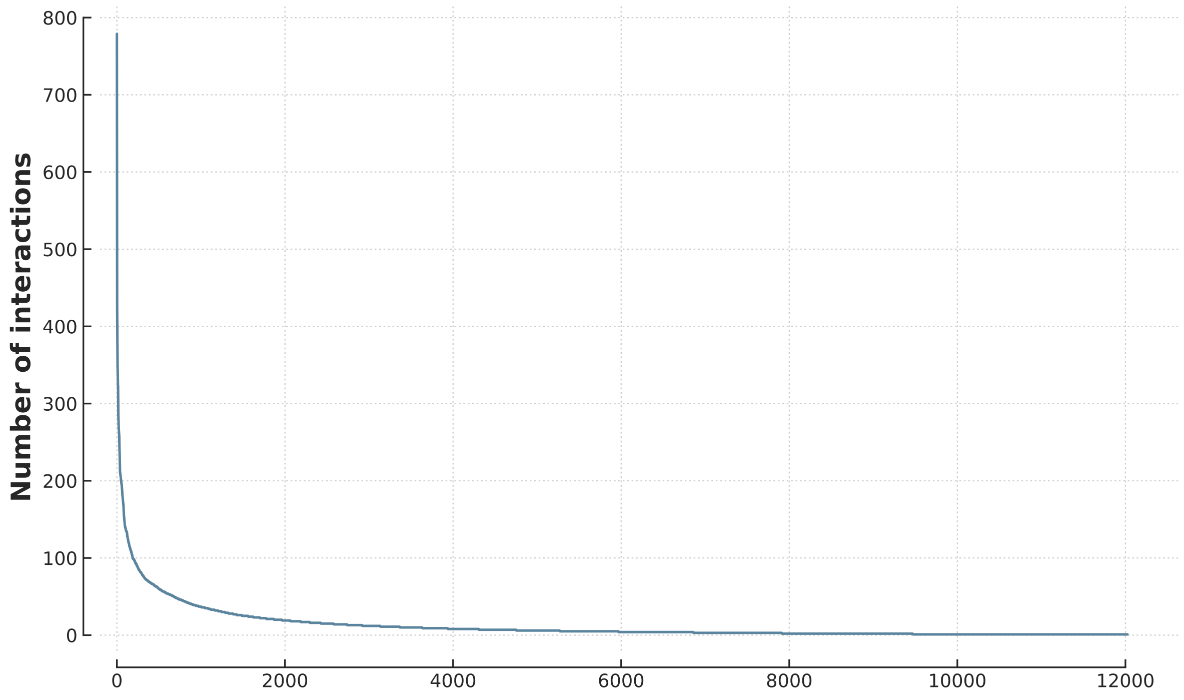

Supplementary Figure 1: Distribution of proteins' degree in IntAct. The exponential decrease is characteristic of a scale-free network.

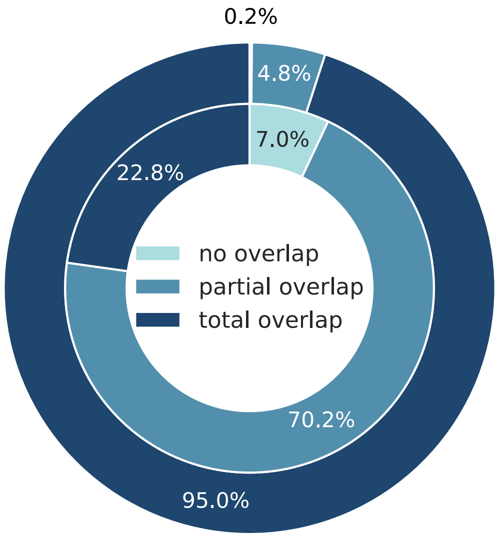

Supplementary Figure 2: The impact of train/test splitting strategies on protein-level overlap. The common splitting strategy is to allocate pairs randomly (outer ring) while here we set aside proteins for testing (inner ring). Dividing the training and testing sets conventionally (using, for example, the popular *scikit-learn* library) resulted in almost all pairs (95%) in the testing set sharing at least one protein with the training set, which may lead to overestimating performances(Park and Marcotte 2012).

Reporting sheet B4PPI-Human: XGBoost

PR and ROC curves on T1* and T2**

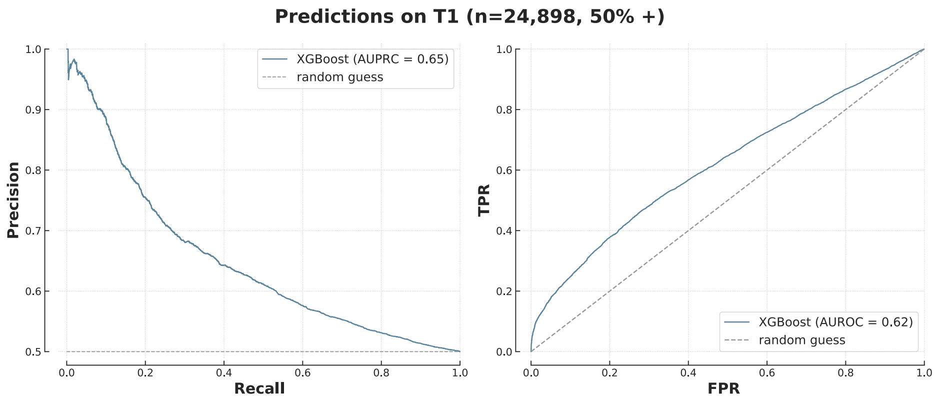

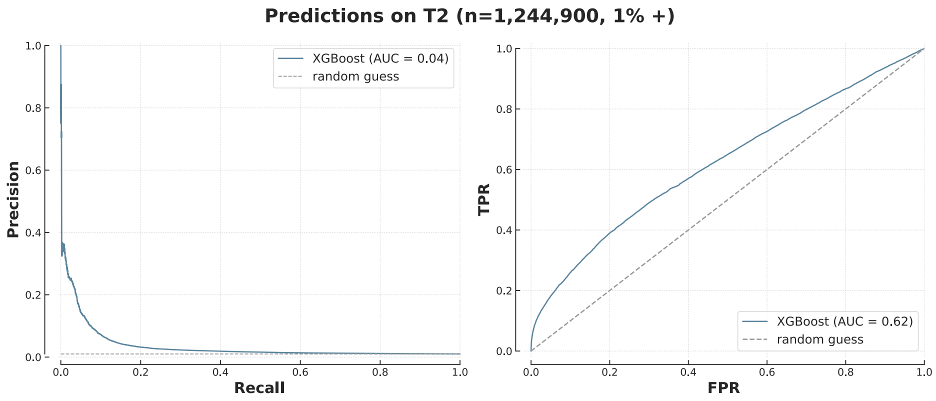

* First testing set, used to compare models on an independent set and investigate protein-level overlap.
** Second testing set, used to assess generalisation on an imbalanced dataset (only 1% of positive examples).

Impact of protein-level overlap

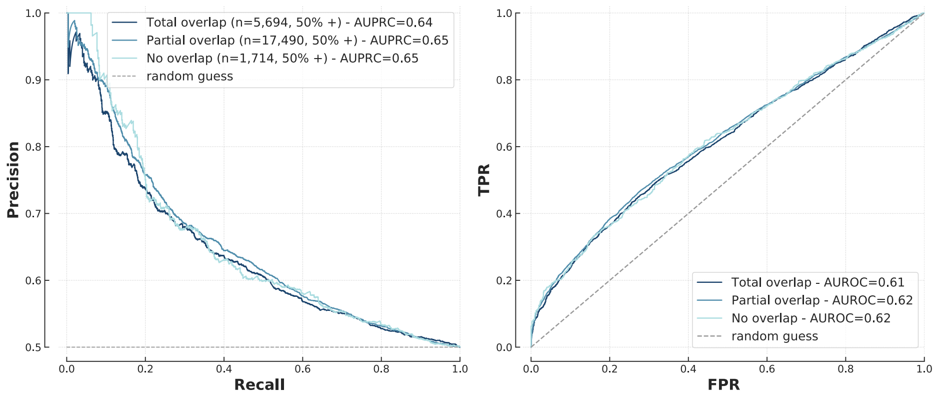

	Running time	Memory	Energy used	Carbon footprint (UK)
Training once	30s	Negligible	< 0.01 kWh	0.002 gCO_2e
Training incl. hyperparameters tuning	22min	Negligible	< 0.01 kWh	1 gCO_2e
Inference	<1s	Negligible	~0	~0

Supplementary Figure 3: Performance sheet of XGBoost on B4PPI-Human.

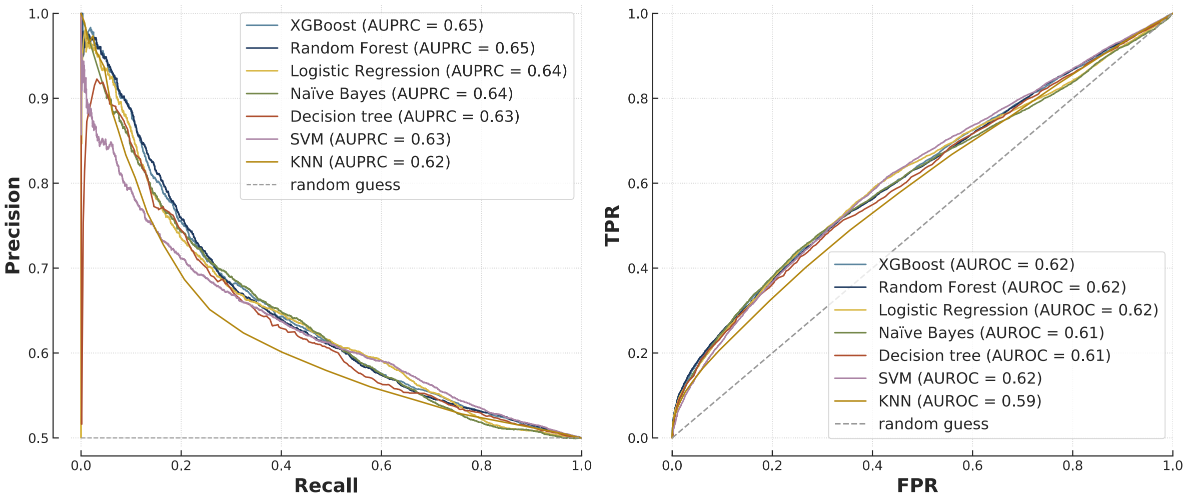

Supplementary Figure 4: Comparison of a broader range of FG-based models on T1.

	coef	std err	z	P>|z|	[0.025	0.975]
RNAseqHPA	0.0196	0.008	2.526	0.012	0.004	0.035
tissueHPA	-0.0380	0.024	-1.576	0.115	-0.085	0.009
tissueCellHPA	0.0617	0.024	2.614	0.009	0.015	0.108
subcellularLocationHPA	0.0144	0.006	2.235	0.025	0.002	0.027
bioProcessUniprot	0.3038	0.011	27.377	0.000	0.282	0.326
cellCompUniprot	0.2650	0.007	37.125	0.000	0.251	0.279
molFuncUniprot	0.0329	0.008	4.353	0.000	0.018	0.048
domainUniprot	0.1696	0.012	14.470	0.000	0.147	0.193
motifUniprot	0.0235	0.007	3.249	0.001	0.009	0.038
Bgee	0.0196	0.007	2.735	0.006	0.006	0.034

Supplementary Figure 5: Output of the logistic regression on the training set.

Reporting sheet B4PPI-Human: Sequence-based

PR and ROC curves on T1* and T2**

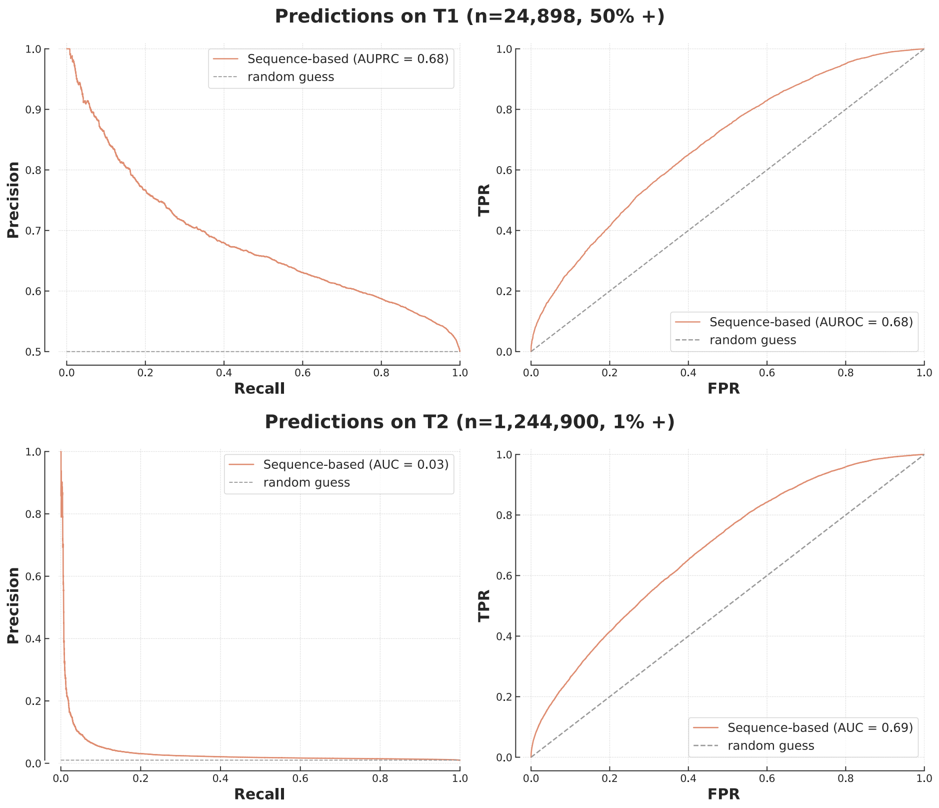

* First testing set, used to compare models on an independent set and investigate protein-level overlap.
** Second testing set, used to assess generalisation on an imbalanced dataset (only 1% of positive examples).

Impact of protein-level overlap

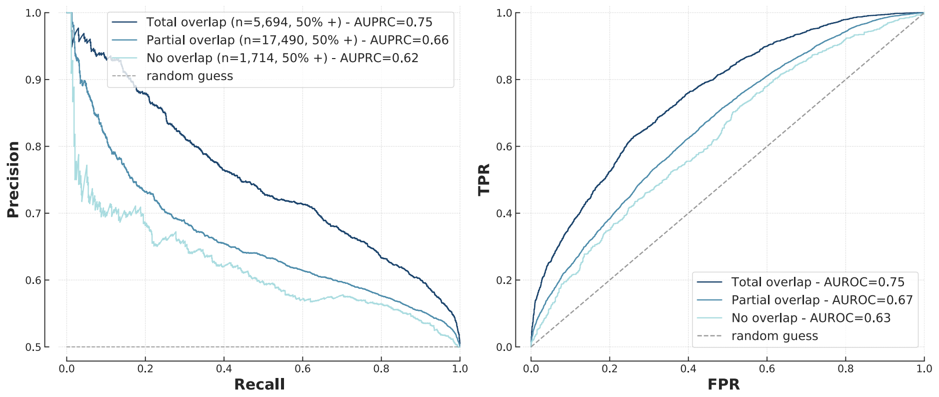

	Running time	Memory	Energy used	Carbon footprint (UK)
Training once	1h10	15 GB	0.75 kWh	189 gCO_2e
Training incl. hyperparameters tuning	>100h	>1.5 TB	> 75 kWh	> 18.9 $kgCO_2e$
Inference (T1+T2)	44min	6 GB	0.50 kWh	115 gCO_2e

Number of (trainable) parameters: 1.6m

Supplementary Figure 6: Performance sheet of the sequence-based model.

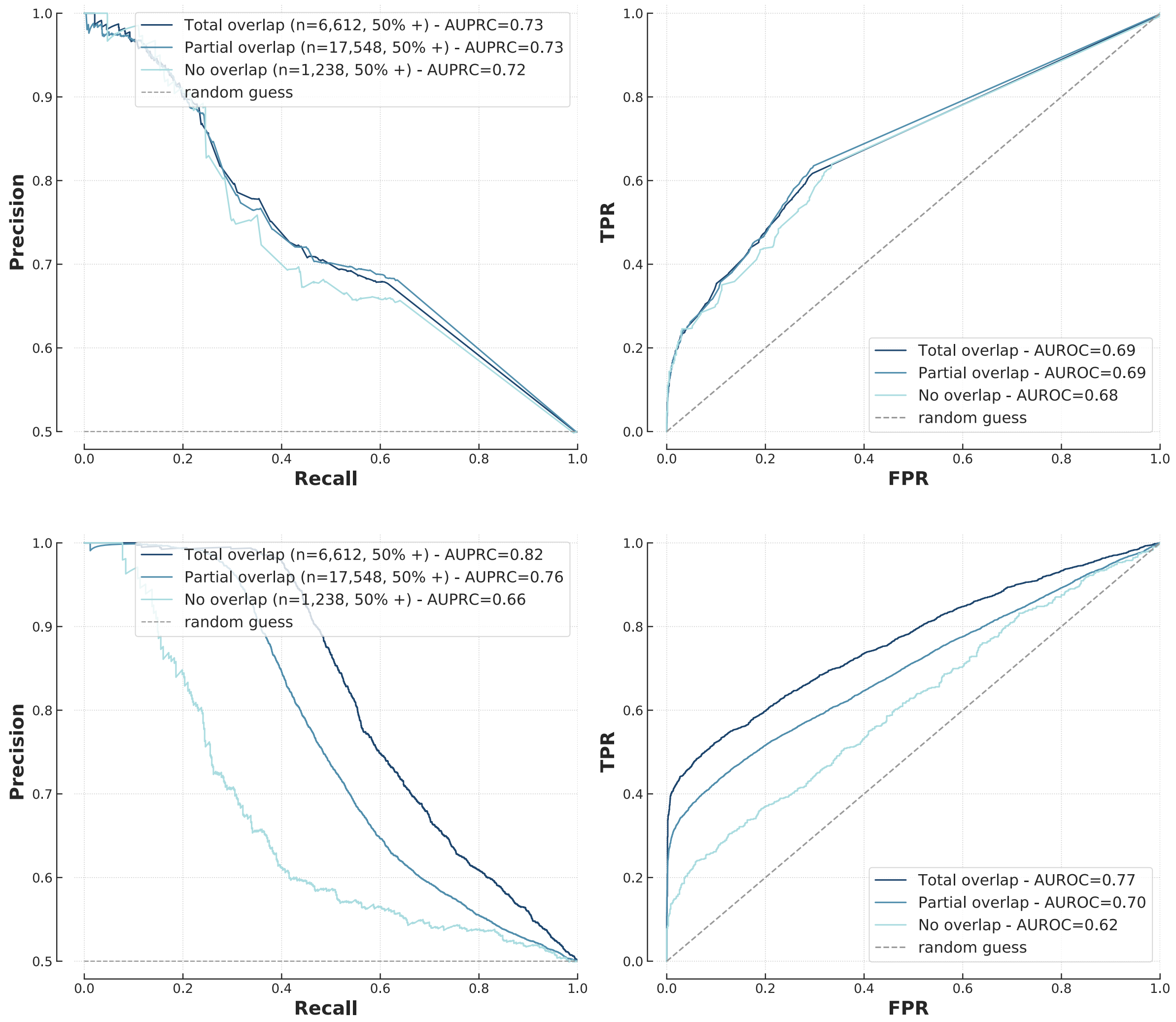

Supplementary Figure 7: Impact of protein-level overlap on the yeast dataset for XGBoost (top, FG-based) and the sequence-based model (bottom).

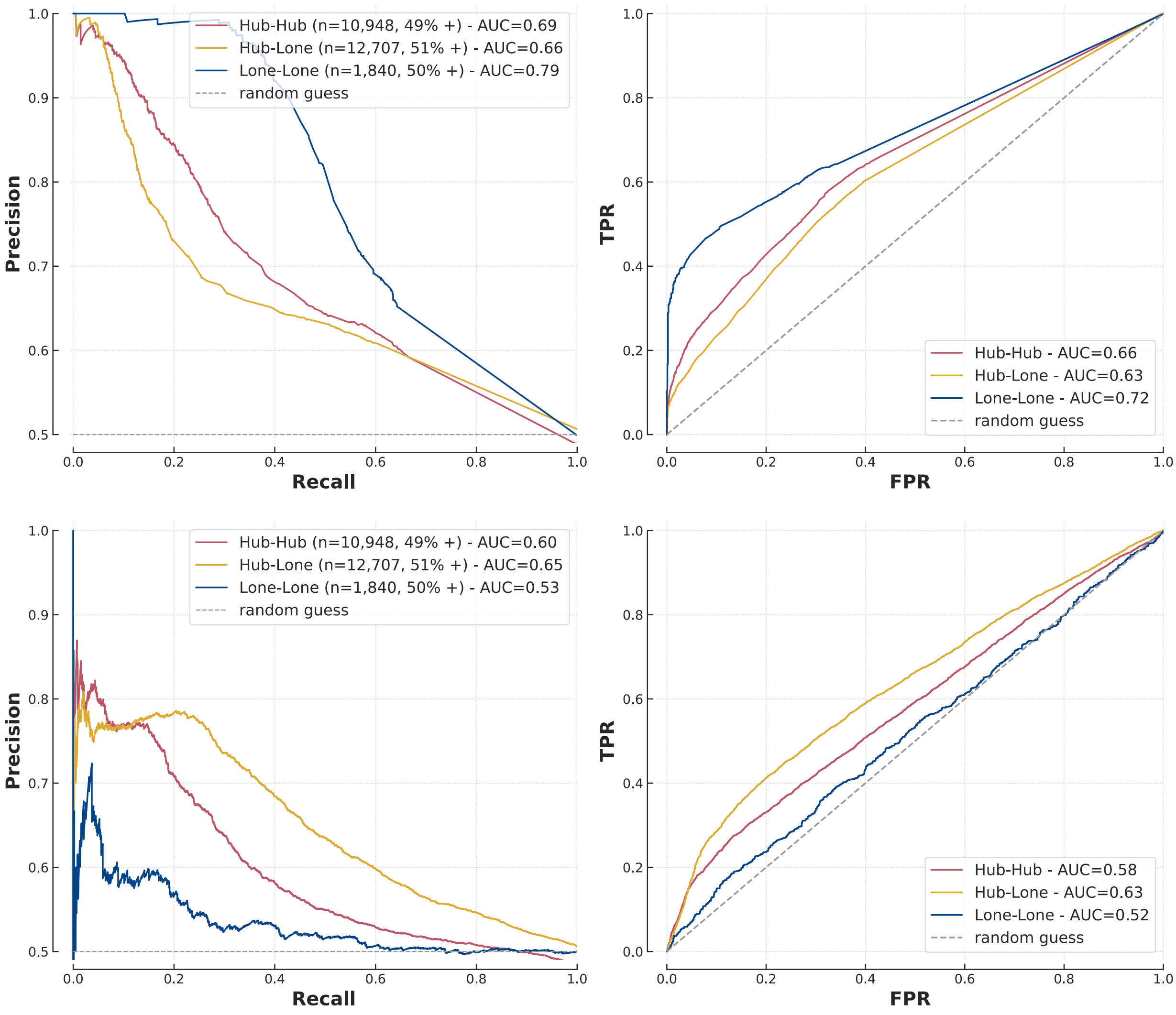

Supplementary Figure 8: Impact of hubs for FG-based (XGBoost, top) and sequence-based (bottom) model on yeast interactions.

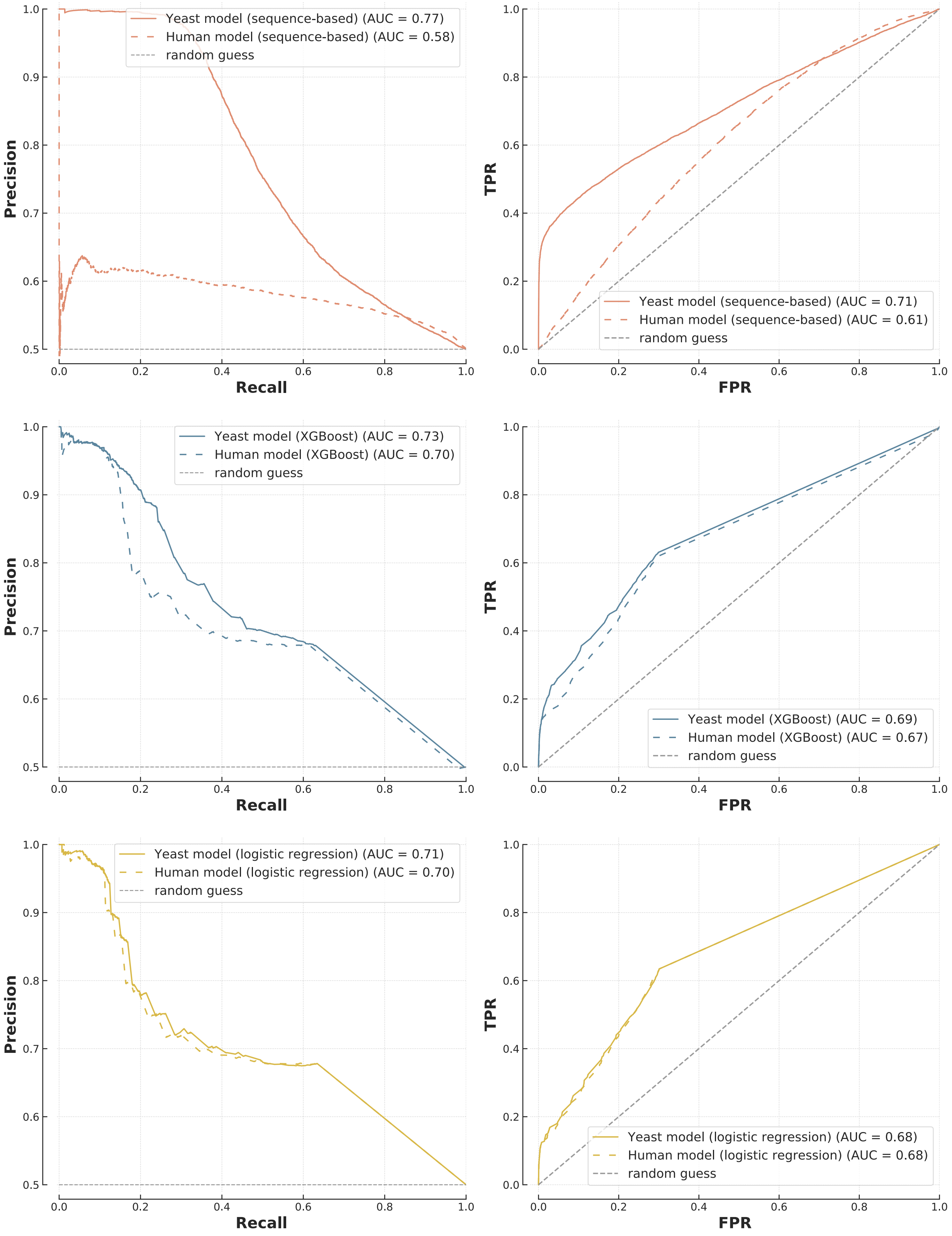

Supplementary Figure 9: Cross-species predictions. Models trained on human PPIs (dotted lines) and yeast PPIs (solid lines) were used to make predictions on the yeast testing set (n=25,398, 50% positive). The top plot is the sequence-based method, the others the FG-based ones (XGBoost in the middle, logistic regression at the bottom).

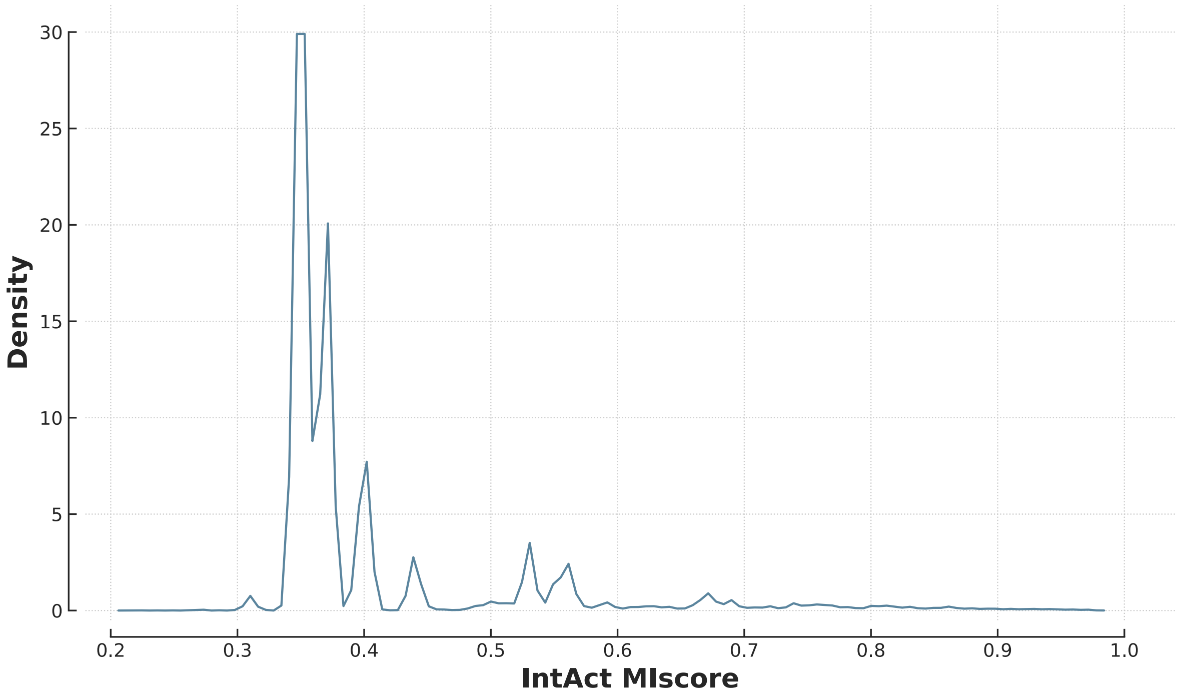

Supplementary Figure 10: distribution of IntAct's MIscore in the yeast dataset.

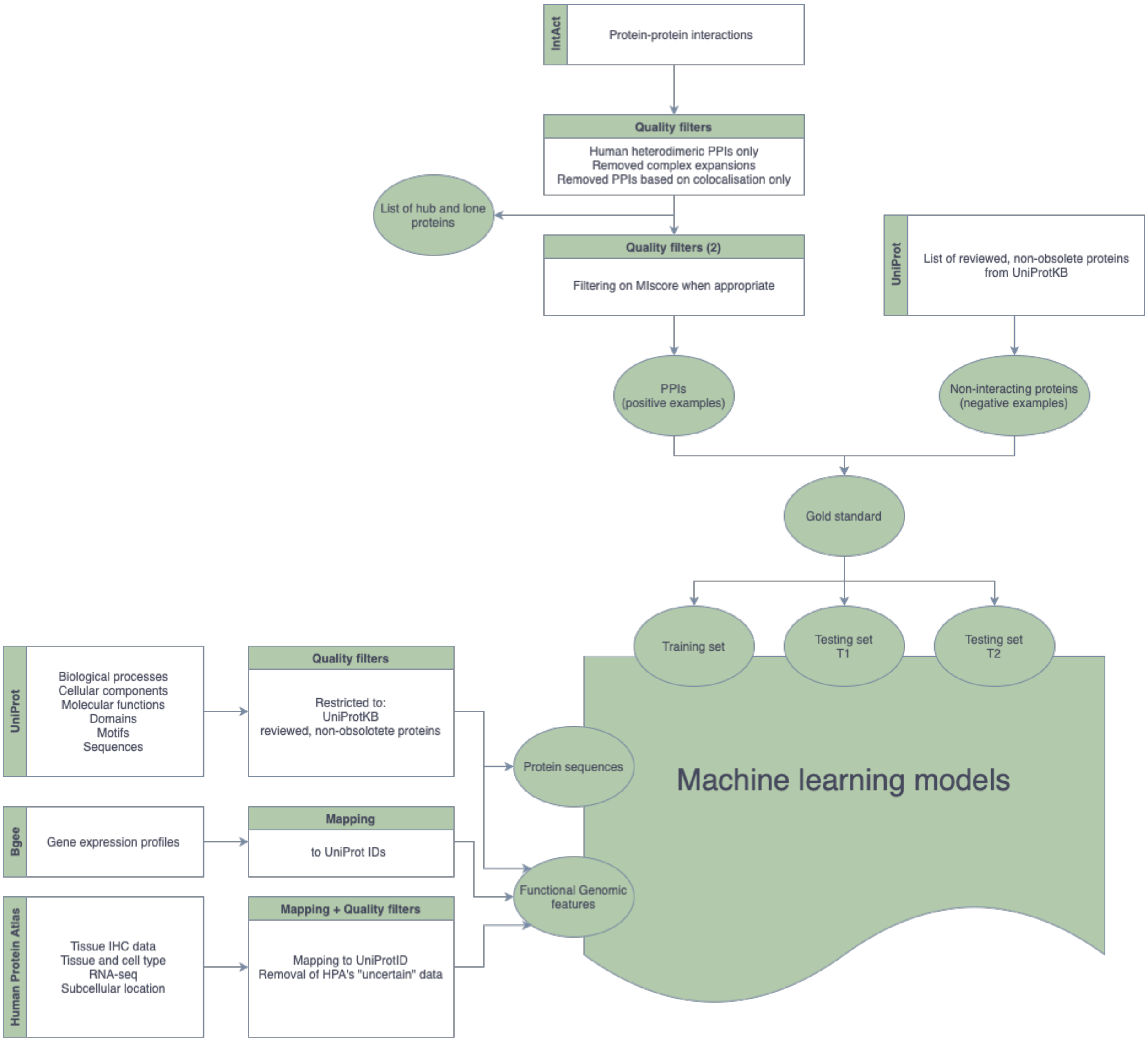

Supplementary Figure 11: Diagram summarising the benchmarking pipeline.

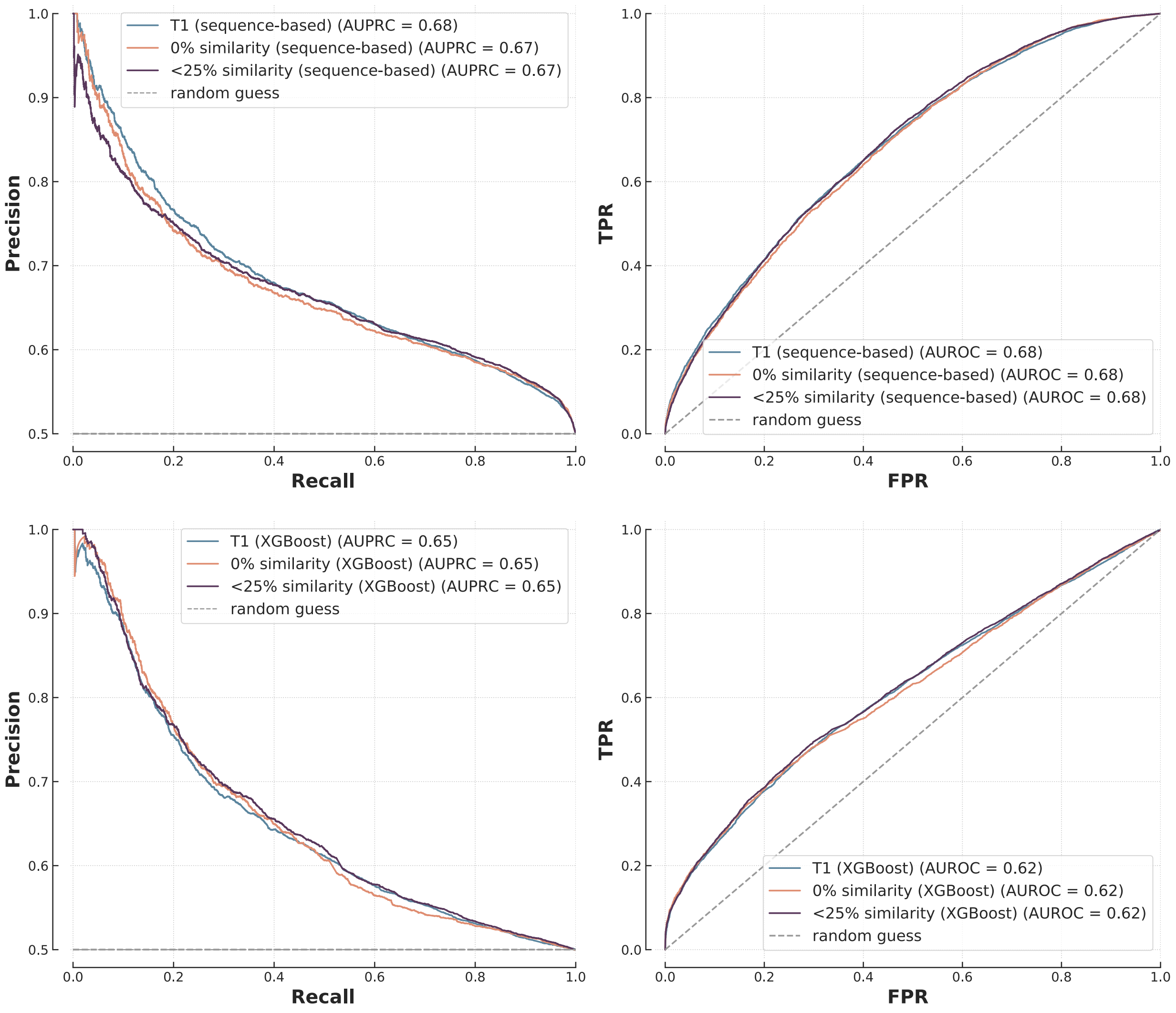

Supplementary Figure 12: Comparison of the performance of the sequence-based model (top) and FG-based XGBoost (bottom) on T1 and the two additional testing sets restricting sequence similarity between the training and testing sets. Both models are unaffected by sequence homology.

Supplementary Tables

Supplementary Table 1: Sample size in B4PPI.

Set	Number of examples (% of positive)	
	B4PPI-Human	Yeast dataset
Training	106,662 (50%)	60,738 (50%)
T1	24,898 (50%)	25,398 (50%)
T2	1,244,900 (1%)	N/A

Supplementary Table 2: Sample size in each category in the dataset used to investigate networks topology.

Type of interaction	Number of pairs	% of PPIs
Hub-hub	27,580	50.69 %
Hub-lone	19,205	49.70 %
Lone-lone	3,011	45.67 %

Supplementary Table 3: Details of the features used for B4PPI-Yeast.

Feature	Number of different annotations	Missing values (/6,721)	Source
Biological processes (GO)	3,114	1,510	UniProt (The UniProt Consortium 2021)
Cellular components (GO)	820	839	UniProt
Molecular functions (GO)	2,079	2,242	UniProt
Domains	606	5,135	UniProt
Motifs	181	6,273	UniProt
Sequence	N/A	0	UniProt

Supplementary Table 4: Optimal parameters for the models trained on B4PPI-Human

Algorithm	Missing data imputation	Scaling	Optimal hyperparameters
Logistic Regression	Yes (mean)	Yes	Penalty = none, tol = 0.0001
XGBoost	No	No	colsample_bytree = 0.8059, learning_rate = 0.00002186, max_depth = 29, min_child_weight = 25, n_estimators = 116, subsample = 0.4595
Decision Tree	Yes (mean)	No	Criterion = entropy, min_samples_split = 895, splitter = random
SVM	Yes (mean)	No	C = 1, degree = 3, gamma = scale, kernel = rbf (default values were used due to long runtime)
Random Forest	Yes (mean)	No	Criterion = gini, max_features = log2, min_sample_split = 487, n_estimators = 336
KNN	Yes (mean)	No	Algorithm = brute, leaf_size = 53, n_neighbors = 35, p = 2, weights = uniform
Naïve Bayes	Yes (mean)	No	N/A
Sequence-based Siamese architecture	N/A	N/A	Batch size = 200, gradient_clip_val = 10, RNN = bidirectional GRU, output = linear, hidden size = 512, n_layers = 1, learning rate = 0.001 (GRU) and 0.0001 (output)

Supplementary Table 5: Optimal parameters for the models trained on the yeast dataset.

Algorithm	Missing data imputation	Scaling	Optimal hyperparameters
Logistic Regression	Yes (mean)	Yes	Penalty = none, tol = 0.0001
XGBoost	No	No	colsample_bytree = 0.7087, learning_rate = 0.00001129, max_depth = 26, min_child_weight = 3, n_estimators = 244, subsample = 0.9318
Naïve Bayes	Yes (mean)	No	N/A
Sequence-based Siamese architecture	N/A	N/A	Batch size = 200, gradient_clip_val = 10, RNN = bidirectional GRU, output = linear, hidden size = 512, n_layers = 1, learning rate = 0.001 (GRU) and 0.0001 (output)
